# Molecular and energetic basis of histidine switch dynamics in respiratory complex I

**DOI:** 10.1002/pro.70720

**Published:** 2026-07-12

**Authors:** Erik Endres, Mahdi Torabi, Mai Jousmäki, Kim Vy Huynh, Cristina Pecorilla, Oleksii Zdorevskyi, Volker Zickermann, Vivek Sharma

**Affiliations:** ^1^ Department of Physics University of Helsinki Helsinki Finland; ^2^ Institute of Biochemistry II University Hospital, Goethe University Frankfurt am Main Germany; ^3^ Centre for Biomolecular Magnetic Resonance, Institute for Biophysical Chemistry Goethe University Frankfurt am Main Germany; ^4^ HiLIFE Institute of Biotechnology University of Helsinki Helsinki Finland

**Keywords:** bioenergetics, enhanced sampling simulations, hybrid QM/MM, mitochondrial respiration, proton pumping

## Abstract

The respiratory complex I in mitochondria and bacteria drives the two‐electron reduction of quinone to pump protons across the membrane. The molecular basis of this catalytic reaction remains enigmatic despite significant progress in structural characterization of the complex. A highly conserved histidine residue in the distal antiporter‐like subunit of its membrane domain has been shown to undergo conformational changes in molecular simulations and cryo‐EM structures. However, the function of histidine switch dynamics and the energetics of its conformational transitions remain unclear. Here, by applying enhanced sampling classical molecular dynamics simulations, we evaluate the energetics of the histidine switch dynamics and demonstrate that it is coupled to the tautomeric state of histidine and to the charge state of lysine residues ca. 10 Å apart, which cause hydrogen bond restructuring and stabilize the histidine residue in specific conformations. Hybrid QM/MM metadynamics‐based free energy simulations show that the histidine switch participates in gated proton transfer and may function as a proton confurcation device in complex I and related proteins.

## 1 INTRODUCTION

**FIGURE 1:**
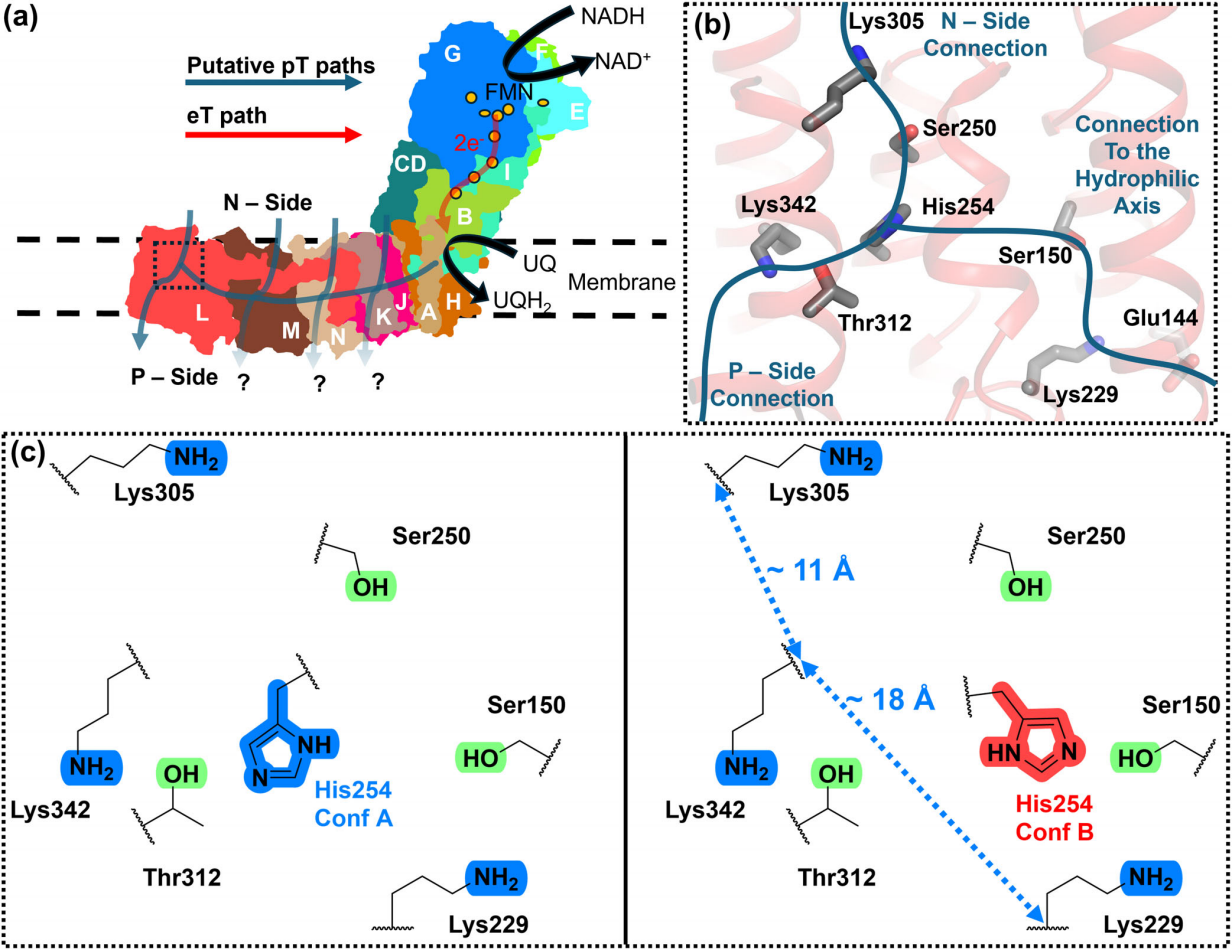
Architecture of RCI and its histidine molecular switch. (a) Bacterial RCI from *Escherichia coli*, PDB 7P7C (Kravchuk et al. 2022). Oxidation of NADH to NAD^+^ provides 2e^−^ that reduce UQ to UQH_2_. This redox reaction triggers a cascade of proton transfers, ultimately pumping protons from the negative (N) side to the positive (P) side. The electron transfer (eT) path and the putative proton transfer (pT) paths are marked in red and blue arrows, respectively. The proton pumping routes towards the P side that remain unclear are denoted with “?”. The bacterial RCI subunits are identified with single letters (e.g., L for subunit NuoL, *E. coli* RCI nomenclature. NuoCD is a fusion of the two “classical” subunits C and D). (b) His254 of NuoL connects to three putative proton transfer routes: the hydrophilic axis (right), the proton uptake site towards N side (top) and the proton release site towards P side (bottom). (c) Schematic of relevant protic residues along the putative paths (panel b) with the distances between lysine residues highlighted. His254 in its Nδ tautomeric state is shown in its two conformations (Conf A/left and Conf B/right) for representational purposes (see text for more details).

Mitochondrial ATP generation by ATP synthase (complex V) requires an electrochemical gradient of protons between the two sides of the inner mitochondrial membrane (Mitchell 1961). This difference in proton concentration is generated by the respiratory complexes I, III, and IV, which effectively function as molecular proton pumps (Wikström et al. 2023), transferring protons only in one direction; that is, from the negatively charged side (N‐side) of the membrane to its positive side (P‐side). Identifying these pumping mechanisms and how their directionality is ensured remains a challenge to this day (Kaila et al. 2008; Yang and Cui 2011). The ~1 MDa Respiratory Complex I (RCI) is the largest complex within the mitochondrial respiratory chain and typically consists of 14 core subunits and around 31 auxiliary subunits (Parey et al. 2020). The bacterial RCIs are relatively smaller in size and consist mainly of the core subunits that form a minimal redox‐driven proton pumping unit. These subunits can be divided into two domains, a solvent‐exposed arm and a membrane‐embedded section (Figure 1a). NADH binds near the FMN binding site and supplies electrons for the reduction of ubiquinone (UQ) at the junction of the two arms (Figure 1a). The latter triggers the pumping of protons throughout the membrane arm, generating the proton gradient for ATP synthesis. There is a general agreement on the pathway of electrons within the complex; FeS clusters, as seen in high‐resolution structures, clearly mark the path from the NADH oxidation site towards the UQ reduction site (Sazanov 2023; Verkhovskaya et al. 2008). The pathways for proton transfer, however, remain dubious (Figure 1a). Some of the key questions are how substrate protons and pumped protons are channeled, how protons travel within the membrane‐embedded subunits, which subunits are involved in the pumping, and above all, what is the molecular basis of the redox‐coupled proton pumping catalyzed by RCI (Djurabekova et al. 2024).

The three membrane‐bound subunits NuoL, NuoM, and NuoN (Figure 1a) that are typically associated with proton pumping (Amarneh and Vik 2003; Euro et al. 2008; Nakamaru‐Ogiso et al. 2010) are homologous to each other and share their architecture with the ancient Multiple resistance and pH adaption (Mrp) cation/proton antiporters (Swartz et al. 2005). Among these, NuoL (or homologous MrpA in Mrp antiporter) displays several anomalous sequence features (Djurabekova et al. 2020; Mathiesen and Hägerhäll 2002) including a strictly conserved histidine His254 (*E. coli* RCI numbering), which was found to undergo protonation‐dependent conformational changes in molecular dynamics (MD) simulations of bacterial RCI (Djurabekova et al. 2020). This histidine, which is positioned on a flexible α‐helical segment, is uniquely situated at the junction of three putative proton transfer pathways; connecting to the hydrophilic axis that runs through all three antiporter‐like subunits via Lys229 of a Lys‐Glu pair, to a potential proton uptake path from the N‐side via Lys305, and to a potential proton release path through Lys342 towards the P‐side (Figure 1b). All three lysine residues are strictly conserved, and biochemical analyses have revealed their functional significance (Amarneh and Vik 2003; Euro et al. 2008; Nakamaru‐Ogiso et al. 2010). High‐resolution cryo‐EM structures of Mrp antiporter demonstrated that histidine adopts two different conformations (Lee et al. 2022): conformation A, pointing towards Lys342, and conformation B, pointing towards the conserved Lys‐Glu‐pair. In conformation A, the histidine is within a hydrogen bonding distance to Thr312, whereas in conformation B, it establishes a hydrogen bond to Ser150 (Figure 1c). Extensive site‐directed mutagenesis and computer simulations of Mrp antiporter further confirmed the functional importance of the histidine conformational dynamics in ion transport (Pecorilla et al. 2025). Building upon this data, we have proposed a model for the histidine switch dynamics in RCI (Pecorilla et al. 2025). Here, by performing atomistic MD simulations on the proton pumping modules of *E. coli* RCI combined with enhanced sampling techniques, we test this proposed model in a quantitative manner by calculating the energetics of histidine dynamics in different charge and conformational states. The results posit that hydrogen bonding restructuring is central for the histidine switch function. Furthermore, metadynamics‐based hybrid QM/MM free energy simulations on hydrogen‐bonded pathways involving histidine provide insights on the energetics of proton transfer. We propose that the histidine switch can function as an efficient proton confurcation device in respiratory complex I and evolutionarily related proteins.

## 2 RESULTS

### 2.1 Dynamics and energetics of the histidine switch with enhanced sampling simulations

**FIGURE 2:**
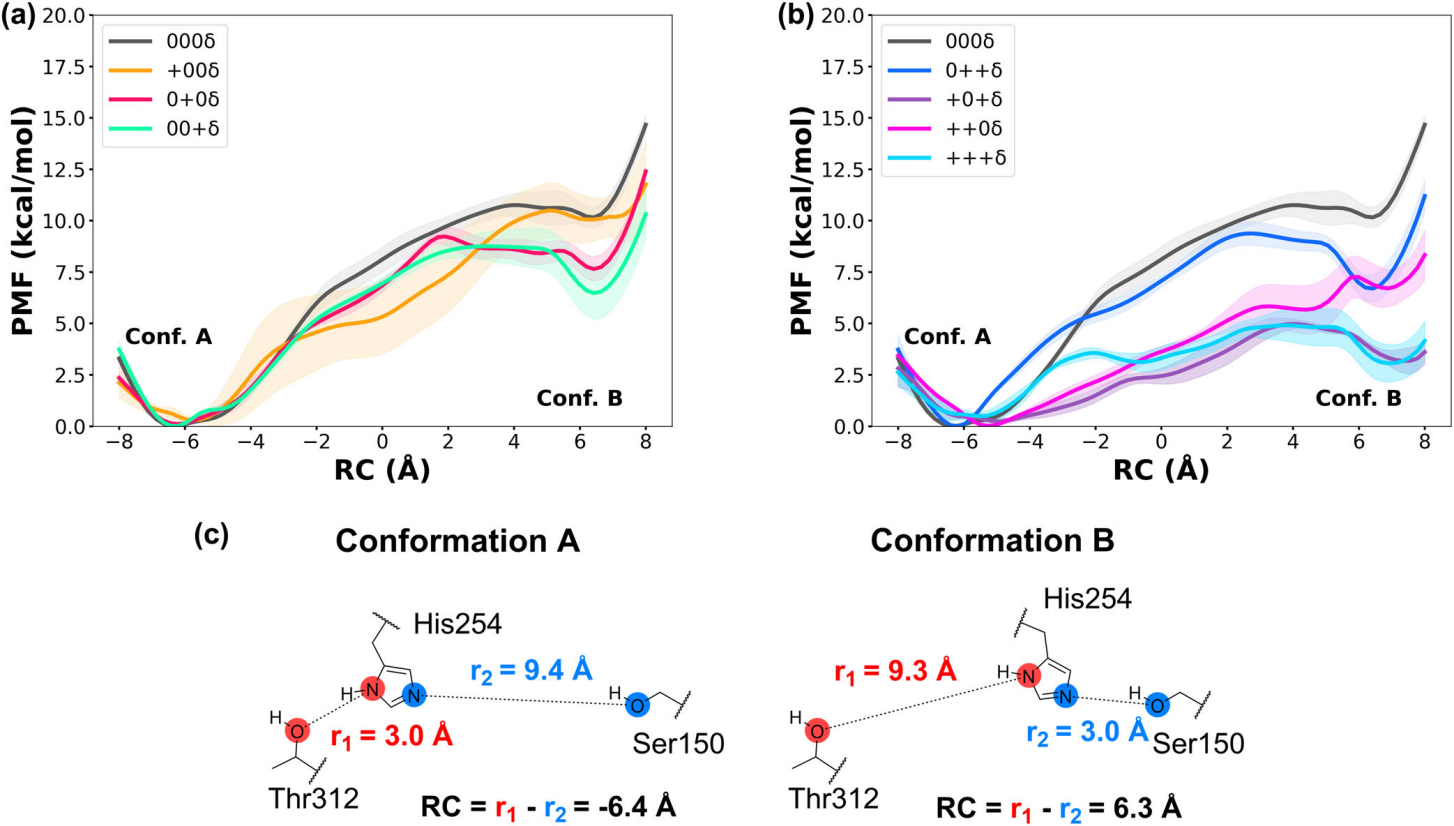
Energetics of δ‐nitrogen protonated His254 sidechain conformational dynamics in different protonation states of conserved lysine residues of the NuoL subunit (Lys342, Lys305, and Lys229). The y‐axis in panels (a) and (b) describes the potential of mean force (PMF, kcal/mol) with respect to the reaction coordinate (RC) on the x‐axis in Å (see methods). Each trace shown is a mean of four simulation replicas, whereas the shaded region describes the standard error of the mean. The short‐hand notation (+00δ) is used to describe the protonation states of lysine residues and histidine. The state +00δ corresponds to protonated Lys342, neutral Lys305, neutral Lys229 and His254 with Nδ‐protonation, in that order. The Nδ and Nε tautomeric states are marked with δ and ε, respectively, whereas imidazolium histidine with p (see Figures S1 and S2). (c) Reaction coordinate (RC) used in AWH simulations. The reaction coordinate was defined as RC = r_1_ − r_2_, so that negative values correspond to being closer to conformation A (left), while positive values correspond to being closer to conformation B (right).

It is known from the modeling and simulations of Mrp antiporter that the protonation states of conserved lysine residues in the MrpA subunit (homologous to NuoL) influence the dynamics of histidine (Pecorilla et al. 2025). We therefore systematically analyzed several different charge states of lysine residues in the NuoL subunit with an enhanced MD simulation sampling method (accelerated weight histogram [AWH]; Lindahl et al. 2014), using a linear combination of His254(Nδ)‐Thr312(Oγ) and His254(Nε)‐Ser150(Oγ) distances as reaction coordinate (RC; see Figure 2 and section 4), and obtained the energetics of the histidine switch in RCI. We find that over a broad range of protonation states, conformation A seems to be favored over conformation B (see Figures 2, S1, and S2, Supporting Information). This agrees with the cryo‐EM structures of *E. coli* RCI where His254 is predominantly observed to be in A conformation or in a conformation close to it. Interestingly, in several high‐resolution structures of bacterial RCI, histidine is found to be stabilized in the A arrangement, in contrast to the B conformation, which is commonly observed in many mitochondrial RCI structures (see Table S1 and Figure S3). We observed that the perturbation of protonation states of selected lysine residues alters the [A]/[B] equilibrium. In contrast to ~10 kcal/mol energy difference in favor of histidine in A conformation in state 000δ (shorthand notation describing protonation states of Lys342, Lys305, Lys229, and His254, and in that order, see also Figure 2), protonation of Lys305 or Lys229 (state 0+0δ or 00+δ) shifts the equilibrium towards B conformation by around 2–3 kcal/mol (Figure 2a). The protonation of Lys342 or Lys229 on top of protonated Lys305 (states ++0δ or 0++δ) also enhances the stability of B conformation. With all three lysine residues protonated (state +++δ) or in state +0+δ, the energy difference between A and B conformations reduces to only about 2–3 kcal/mol, highlighting an important role of Lys229 protonation in enhancing B population (Figure 2). A similar lysine protonation dependent effect on histidine dynamics can be observed in its ε‐nitrogen protonated tautomeric state (Figure S1). Overall, there are only a handful of states when the energetic difference between A and B conformations is in the range of 2–3 kcal/mol.

**FIGURE 3:**
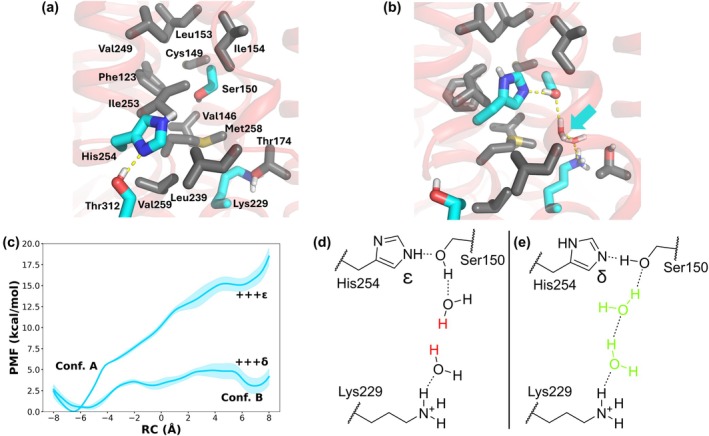
Structural changes upon histidine dynamics and its dependence on tautomeric states. The movement of His254 from A position (a) to B position (b) is blocked by a cluster of hydrophobic residues. The hydrophobic cluster rearranges upon charge‐induced hydration in the region, in part caused by the protonation state changes of surrounding lysine residues (see text). The hydrogen bonds of His254 with Thr312 in A position and with Ser150 in B position are shown (yellow dotted lines), along with the water molecules (cyan arrow) that populate the region upon lysine protonation state changes (panel b). The snapshots shown in panels (a) and (b) are from states +++ε and +++δ, respectively. (c) The PMF profiles from δ‐ and ε‐nitrogen protonated His254 AWH simulations in +++ state. (d, e) Schematic drawing of different hydrogen bond patterns due to the δ‐ and ε‐tautomeric states of His254.

It is noteworthy that changing protonation states of lysine residues ca. 10–20 Å apart (Figure 1) can have a significant influence on the position of His254. To probe what stabilizes (or destabilizes) the A/B conformations, we analyzed the simulation trajectories. When histidine occupies the A position (Figure 2), the region between His254 and Ser150/Lys229 is packed with several bulky lipophilic side chains of Leu153, Ile154, Cys149, Val249, Ile254, Met258, Trp238, Leu239, Val259, and Val146, including the mitochondrial disease locus Phe123 (Hoeser et al. 2025; Pecorilla et al. 2025) (Figure 3). These essentially impart a hydrophobic barrier towards the B conformation. Only when Lys229 (and also Lys305) is modeled protonated, hydration in the region increases, leading to the destabilization of the hydrophobic blockage and establishment of the B conformation of His254 (Figure 3).

### 2.2 Energetics and dynamics of the histidine switch in different tautomeric states

We also find that the tautomeric states of charge neutral histidine (δ‐ or ε‐nitrogen protonated) can have a strong influence on His254 dynamics and energetics (Figures 2, 3, and S1). For instance, in state +++δ, His254 orients to form a stable hydrogen bonding interaction with Ser150 (Figure S4), whereas the other tautomer (ε‐nitrogen protonated) is unstable in the B position (Figure 3c). We rationalize this using the difference in extended hydrogen bonding patterns in the two states involving the ε‐nitrogen of histidine, whereas the Ser150‐His254 hydrogen bond via δ‐nitrogen is likely hindered geometrically as well as sterically. In the δ‐nitrogen protonated state, His254 stabilizes in the B position with the ε‐nitrogen acting as a hydrogen bond acceptor from Ser150, which in turn participates in a stable hydrogen bond network towards protonated Lys229 (Figures 3e and S4). However, when the ε‐nitrogen of His254 is protonated, Ser150 does not act as hydrogen bond acceptor anymore, thereby leading to perturbation in the water‐based hydrogen bonding with Lys229 and destabilization of the B conformation of histidine (Figure 3d). Similarly, in the other protonation states simulated, the histidine switch dynamics is found to be tautomer dependent. In 0+0 (or 000) charge state of lysine residues, His254 with ε‐nitrogen protonated stabilizes in the B position by about 5 kcal/mol in comparison to the δ‐nitrogen protonated state (see Figures 2, S1, and S4).

**FIGURE 4:**
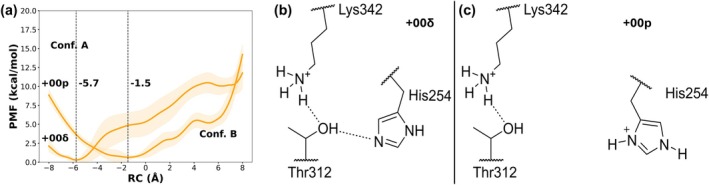
Protonation of neutral His254 causes a shift of the energy minimum within the PMF profile. (a) PMF profiles of histidine switch dynamics in two protonation states. The imidazolium state has its minimum shifted away from A position towards a state in between A and B. Panels (b) and (c) provide molecular and chemical explanations of the shift. With His254 in charge neutral state, it accepts a hydrogen bond from Thr312, which is no longer possible when histidine is doubly protonated. This, as well as the electrostatic repulsion from protonated Lys342, drives the protonated histidine towards the in‐between states (see also Figure S2).

Previous simulation studies on Mrp antiporter showed that the doubly protonated histidine can occupy an intermediate position between the A and B conformations (Lee et al. 2022; Pecorilla et al. 2025). We modeled His254 in its imidazolium state and observed that it preferentially occupies the middle position, especially when Lys342 is protonated (+00), with both A and B positions at least 4 kcal/mol higher in energy (Figure 4a). The effect of charge states of Lys305 and Lys229 on protonated histidine is relatively weak, and His254 occupies a position close to the A conformation, whereas the B position is unfavorable by at least 8 kcal/mol in all states studied (Figure S2). A closer analysis of simulation trajectories reveals that a consecutive charge‐assisted hydrogen bond network can be formed by protonated Lys342, Thr312 and neutral His254 (Figure S4b), explaining why the PMF‐profile is stabilized in the A (or A‐like)—conformation at an approx. RC ~ −6 Å. In this scenario, His254 acts as a hydrogen‐bond acceptor for the hydroxyl group of Thr312, whereas this is no longer possible in the imidazolium state of histidine. With His254 being positioned on a flexible α‐helical segment, it therefore shifts away from the A conformation to an in‐between position at an approx. RC ~ −1.5 Å (Figure 4b,c) or to similar values in other charge states (see Figure S2).

**FIGURE 5:**
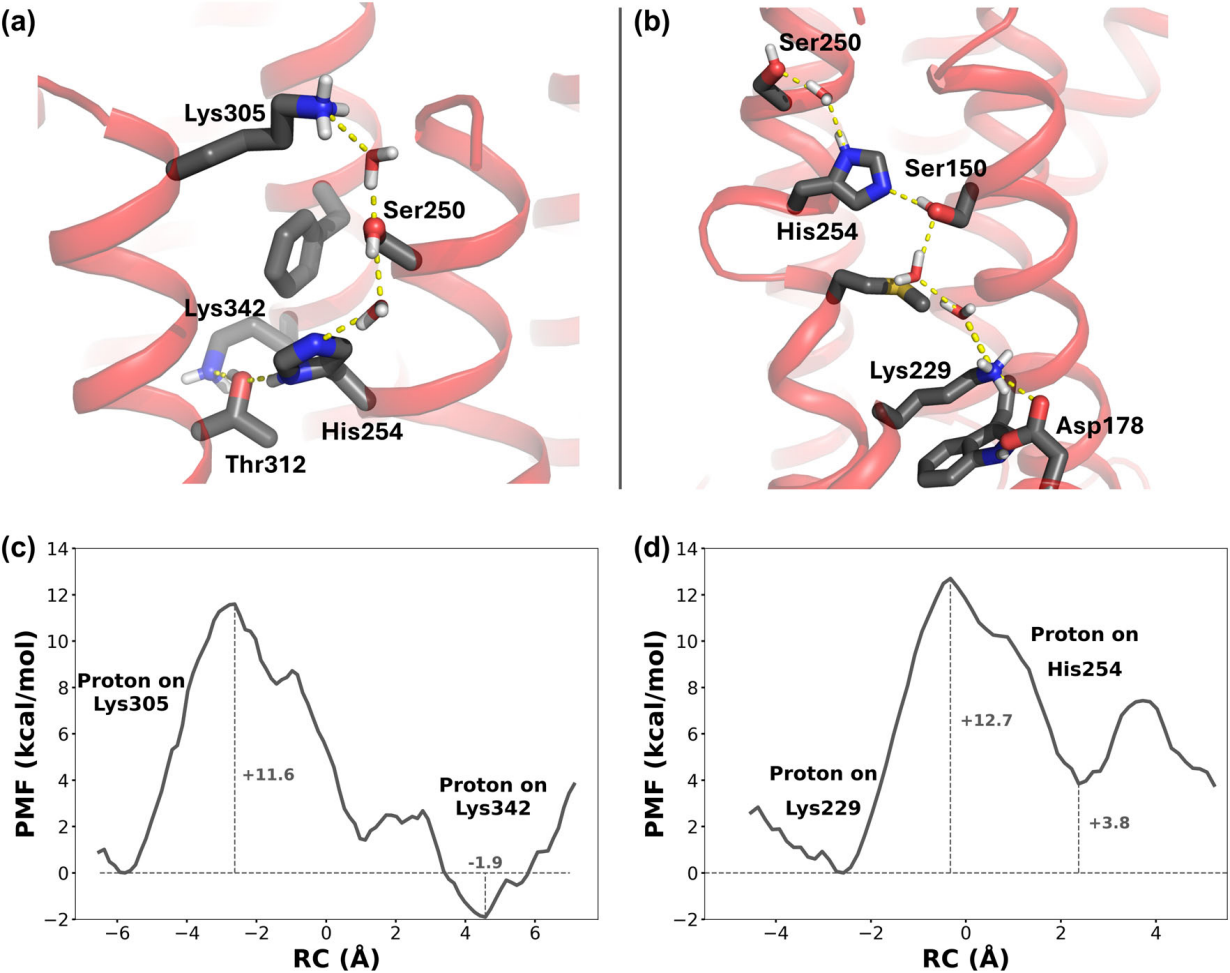
Hydrogen bonded pathways and energetics of proton transfer. Snapshots showcasing the hydrogen bonding patterns observed in AWH‐based classical MD simulations of setups 0+0δ (a) and +++δ (b) with the histidine in conformation A and B, respectively. The hydrogen bonds are shown with dotted lines. Parts of transmembrane helices are not shown for clarity. (c) PMF profile of proton transfer from Lys305 to Lys342 involving histidine in A position (panel a) and (d) from Lys299 to His254 in its B position (panel b) as a function of RC (in Å). The energy profiles shown in panels (c) and (d) are from 26 and 20 ps of well‐tempered metadynamics QM/MM simulations (replica # 1), performed with Glu359 and Asp178 protonated, respectively (see also Figure S5 for PMF data from additional replicates). The PMF profiles are extracted after one full complete sampling of the reaction coordinate space (see other criteria in methods and Figures S13 and S14). See Figure S12 for description on RC.

The RCI histidine switch model (Pecorilla et al. 2025) suggests that His254 acts as a bridging element between the putative proton transfer paths (Figure 1) and that it can minimize the formation of networks that lead to energy loss in terms of proton transfer down the gradient. Figure 5 shows that in conformation A, the charge neutral His254 can bridge the solvent exposed Lys305 with the buried Lys342 via a hydrogen bonding network involving conserved Thr312 and Ser250, and water molecules (see also Figure S4a). This pathway is reminiscent of a putative proton uptake route from the N side of the membrane. Since this His254‐bridged connection is delinked from Lys229 of the Lys‐Glu pair in the central hydrophilic axis, a likely function of the histidine switch is to prevent the premature protonation of anionic species generated upon quinone reduction at the other end of the complex (Djurabekova et al. 2024). Furthermore, when His254 is in its B conformation, it participates in a path from protonated Lys229 (of Lys‐Glu pair) to the δ‐nitrogen of the histidine via two water molecules and Ser150 (Figures 5 and S4c). In this arrangement, the Lys342 and Lys305 remain disconnected, thereby effectively preventing a P‐side to N‐side connection.

### 2.3 QM/MM free energy simulations of proton transfer

To investigate if the observed hydrogen bonded pathways (Figure 5a,b) can catalyze proton transfer, we performed well‐tempered metadynamics QM/MM free energy simulations on the AWH‐derived simulation snapshots (see section 4 and Figure 5a,b). We observed that a proton can be transferred from the cytoplasm facing protonated Lys305 to the buried and charge neutral Lys342 involving histidine in A position (Figure 5c). In two of the three independent simulation replicas, an energetically favorable proton transfer can be observed (see Figures 5c and S5). In the third replicate, only a partial proton transfer occurs from the region around Ser250 to the buried Lys342 (Figures 5a, S5, and S6). Due to the conformational changes near the cytoplasm facing proton donor Lys305, a complete proton transfer is not observed in the time scale of the simulations and as a result an energy minimum at RC ~4 Å is not observed in this replica (Figure S5). Nevertheless, given that two out of the three independent simulations demonstrate a possibility of favorable proton transfer, we envisage this proton transfer step may correspond to the loading of a pumped proton to the NuoL subunit from the cytoplasmic side in response to the quinone reduction reaction ca. 20 nm away. Several lines of evidence suggest a long‐range electron‐proton coupling in RCI (see section 1). For instance, the mutation of Lys342 to alanine in the terminal antiporter‐like subunit ND5 reduces the quinone reductase activity by ~90% (Sato et al. 2013). We suggest that the histidine switch in A position effectively gates the transfer of cytoplasmic proton towards the quinone reduction site, which can cause uncoupling (see Note in Supporting Information).

When the proton transfer between histidine in B position and Lys229 is probed by QM/MM simulations, we find the protonation of His254 to be energetically unfavorable (Figures 5d and S5). In all the three simulation replicas, the product states are found to be unstable by at least ~4 kcal/mol (Figures 5d and S5). Additional conformational and charge changes in protein could make this proton transfer energetically favorable. It is indeed known from classical MD simulations that protonated histidine can stabilize a position in‐between the two conformations A and B (Djurabekova et al. 2020; Pecorilla et al. 2025), but such dynamical effects as well as long‐range electrostatic effects are challenging to account for in the QM/MM approaches, which could bring the energetics favorable for histidine protonation. Therefore, we extended our QM/MM simulations, and this additional sampling improved the energetics of the product state (protonated histidine) and lowered the activation energy barriers in selected simulation replicas (Figure S13). Overall, based on our QM/MM calculations, we suggest that histidine can participate in proton transfer reactions in its A and B conformational states, and at the same time act as a molecular gate to prevent uncoupling.

## 3 DISCUSSION

All proposed models (Djurabekova et al. 2024; Kaila 2018; Sazanov 2023) suggest NuoL is involved in proton pumping by RCI. Being the most distant subunit from the quinone active site (Figure 1), it must possess unique structural elements that enable efficient long‐range coupling between the quinone reduction reaction and proton pumping. The highly conserved His254 is one such feature that may assist in efficient long‐range coupling in RCI (Djurabekova et al. 2020). The histidine residues, which display a variety of charge and tautomeric states in proteins, are known to play key roles in the proton pumping mechanism of other respiratory complexes (Morgan et al. 1994; Popović and Stuchebrukhov 2004). Based on our enhanced sampling simulations and QM/MM approaches, we find that His254 in RCI undergoes protonation‐state dependent conformational dynamics, shifts between putative proton transfer pathways (Figures 1 and S4), and also acts as a proton transfer element. By stabilizing in the A conformation, His254 effectively shuts off the connectivity of the central hydrophilic axis with the proton transfer pathways of the terminal antiporter‐like subunit of RCI, while in the B conformation, it detaches the proton uptake and release routes of the NuoL subunit. Furthermore, in the A position, it participates in proton uptake from the cytoplasmic side, whereas in the B conformation, it can function as a proton carrier from the hydrophilic axis of RCI. Essentially, by switching between A and B conformations, His254 carries protons from the N side and the central hydrophilic axis to a proton release route towards the P side, thereby functioning as an efficient proton confurcation device. Overall, our multiscale simulation data strongly suggest that His254 is in fact a type of molecular switch that is capable of turning proton transfer routes on and off. The protonation states of the surrounding lysine residues (Lys229, Lys305, and Lys342) play a key role in triggering this switch, by rearranging water‐ and amino acid residue‐based hydrogen bond networks. Further experimental testing by site‐directed mutagenesis and cryo‐EM studies will be required to strengthen the proposed model of the histidine molecular switch.

The conservancy of this histidine extends beyond RCI and Mrp antiporter sequences, where it is also found in photosynthetic complex I (Zhang et al. 2020) and energy converting hydrogenase (Ech) (Katsyv and Müller 2022). Intriguingly, it is not conserved in this locus in membrane‐bound hydrogenase (Mbh) (Yu et al. 2018), membrane‐bound sulfane sulfur reductase (Mbs) (Yu et al. 2020), and formate hydrogenlyase (Fhl) enzymes (Steinhilper et al. 2022). However, the possibility of functional compensation by a histidine from another region cannot be slighted. Indeed, in agreement with this view, the Fhl structure from *E. coli* shows a histidine residue (His222, *E. coli* Fhl numbering, PDB 7Z0S) that occupies a similar location and may take up the role of the histidine switch (Figure S7). Further analysis of these putative switches in other protein classes is a subject of future studies.

## 4 COMPUTATIONAL METHODS

The 2.4 Å cryo‐EM structure of *E. coli* RCI (PDB 7P7C) (Kravchuk et al. 2022) was used to construct the simulation model system comprising the five membrane‐bound core subunits (NuoL, ‐M, ‐N, ‐K, and ‐J). A truncated model was prepared to keep the system size tractable and achieve enhanced simulation sampling in different chemical and conformational states (see below). Previously, truncated models of RCI have been simulated successfully providing functional insights (Galemou Yoga et al. 2020; Lasham et al. 2023; Nishida et al. 2025). The missing loops of NuoL (440–453, 514) and NuoN (437–445) were added using Modeller (Šali and Blundell 1993) and in addition the missing sidechain of Glu359 was modeled. Protonation states were defined using PropKa (Søndergaard et al. 2011) (Table S2), with the exception of His254, Lys305, Lys342, and Lys229, which were simulated also in their alternative protonation states leading to in total of 24 different simulation setups (see Table S3). The OPM‐aligned (Lomize et al. 2012) protein complex was embedded in a lipid bilayer composed of 75% POPE, 20% POPG, and 5% dianionic cardiolipin, obtained using CHARMM‐GUI (Jo et al. 2008). The membrane‐protein system was solvated in a TIP3P water box with a salt concentration of 0.15 M Na^+^/Cl^−^. The CHARMM36 forcefield (Huang and MacKerell 2013) was used to describe the protein, lipids, ions, and solvent. The polarization effects, which can affect protein dynamics and energetics, were not explicitly taken into account (see, e.g., Leontyev and Stuchebrukhov 2010; Lamoureux 2003). The model system comprised approx. 300,000 atoms (Figure S8).

All classical atomistic MD simulations were performed with Gromacs‐2022.4 (Abraham et al. 2015). Prior to production simulations, the system was energy minimized with a harmonic constraint of 2000 kJ mol^−1^ nm^−2^ on the protein heavy atoms. The resulting structure was equilibrated with a multi‐step procedure, in analogy to prior literature (Lee et al. 2022): First, the temperature was equilibrated to 310 K for 100 ps in an NVT ensemble using the v‐rescale thermostat (Bussi et al. 2007). During this step, the constraints of the system were extended to the phosphorus atoms of lipids to prevent their movement in the z‐direction. In the second step, the system was equilibrated in an NPT ensemble for 1 ns using the Berendsen barostat (Berendsen et al. 1984) (1 atm) and a v‐rescale thermostat (Bussi et al. 2007) (310 K) with the restraints from the phosphorus atoms removed. As a last equilibration step, all restraints were removed, and the system was simulated for 10 ns.

For the production simulations, the Parrinello‐Rahman barostat (Parrinello and Rahman 1981) and the Nose‐Hoover thermostat (Evans and Holian 1985) were employed to maintain a pressure of 1 atm and 310 K temperature, respectively. The long‐range electrostatics were accounted for with PME (Darden et al. 1993), and a time step of 2 fs was achieved using the LINCS algorithm (Hess et al. 1997) implemented in Gromacs. A 12 Å cutoff for non‐bonded interactions was used. Three 500 ns unbiased MD simulations were performed for each state to obtain starting coordinates for the AWH (Lindahl et al. 2014) enhanced sampling simulations with default parameters. The biased reaction coordinate for the AWH simulations is described in Figure 2. Four walkers were employed in each AWH run, and four replicas were simulated for each protonation state considered. The convergence of the AWH simulation was achieved in ca. 200 ns (see Figures S9 and S10). The total unbiased MD simulation sampling is 39 μs, and the AWH simulations correspond to ca. 20 μs, totaling ~59 μs of atomistic simulation sampling. The simulation data were analyzed using VMD (Humphrey et al. 1996), the Gromacs toolbox (Abraham et al. 2015), MDAnalysis, and its WaterBridgeAnalysis module (Gowers et al. 2016; Michaud‐Agrawal et al. 2011). Figures were produced using VMD and PyMol (Schrodinger 2015).

Snapshots for putative proton transfer pathways (Figure 5a,b) were extracted from the AWH simulation trajectories to perform the well‐tempered metadynamics‐based QM/MM free energy calculations. The QM region consisted of the amino acid residues and water molecules displayed in Figure 5 as well as the surroundings (see Table S4). For QM/MM simulations, NAMD 2.14 (Phillips et al. 2020) and Orca 6.0 (Neese et al. 2020) software were used. For the MM region, the CHARMM36 forcefield (Huang and MacKerell 2013) was employed, and the QM region was described by DFT with B3LYP (Becke 1993; Lee et al. 1988; Stephens et al. 2002; Vosko et al. 1980) functional and def2‐SVP (Weigend and Ahlrichs 2005) basis set. The dispersion effects were accounted for by using the D3BJ correction (Grimme et al. 2010; Grimme et al. 2011). The link‐atom approach was used (link atom placed between CA and CB atom of the amino acid residues) together with the additive electrostatic embedding framework as implemented in NAMD.

The QM/MM model system with PBC implemented was energy minimized with 300 steps, followed by 1 ps of equilibration. Production runs of ~30 and ~20 ps with well‐tempered metadynamics for setups A and B, respectively (see Figure 5a,b) were initially performed by selecting the same protonation states for all residues as applied in the classical MD simulations (see above). The energy barriers obtained in these initial simulations were high and no proton transfer activity was observed (not shown). Therefore, to trigger protonation dynamics, three independent production replicas were initiated from the same starting coordinates, but with different random seeds, by protonating Glu359 and Asp178 (see Table S4). Glu359 is not fully resolved (sidechain atoms missing) in the cryo‐EM model (PDB 7P7C) suggesting its conformational mobility. Given its proximity to the solvent phase at the N side of the membrane, it is likely to be anionic. However, our classical MD simulations showed its proximity to the key lysine residue Lys305 and stabilization of a hydrogen bond between the two (Figure S11), suggesting that Glu359 could act as a proton transferring element to Lys305. Therefore, its charge neutral state was modeled in all subsequent QM/MM simulations of histidine in A state. Asp178 resides on the central hydrophilic axis of complex I that is known to catalyze proton transfer in coupling to the redox reactions at the quinone binding site (see, e.g., Djurabekova et al. 2024; Sazanov 2023). Proton affinity estimates on MD simulation data supported high pKa of Asp178 (Figure S11). Therefore, it was modeled charge neutral in subsequent QM/MM calculations to trigger the protonation dynamics in B conformation of histidine. The notion is in line with the proton‐injection driven proton pumping mechanism (Parey et al. 2021) where loading of a proton on one site triggers transfer of another proton due to electrostatic repulsion.

The well‐tempered metadynamics parameters hill height, hill width, and Δ*T* were 0.3 kcal/mol, 0.3 Å, and 4808 K, respectively (see Torabi 2026). A cutoff of 12 Å was applied for non‐bonded interactions with switching function at 10 Å. The long‐range electrostatics was treated with PME, as implemented in NAMD (Essmann et al. 1995). The temperature (310 K) was maintained with Langevin thermostat implemented in NAMD. The reaction coordinate to bias proton transfer was applied as discussed in Figure S12. The *colvars* module 2020‐07‐07 (Fiorin et al. 2013) was used to define the reaction coordinate.

It is challenging to estimate the convergence of well‐tempered metadynamics simulations, especially those that are performed in the computationally intensive framework of QM/MM (Hsu et al. 2016; Sun et al. 2017) and with larger QM region size (~170 atoms, Table S4) at B3LYP DFT level. With such computational expense, multiple transitions of RC on free energy surface are difficult to achieve. Therefore, we decided to extract the PMF data after the reaction coordinate sampled the defined path, and also by observing that hill heights were sufficiently small (~0.05 kcal/mol) and the PMF profiles were stabilized (Figures S6, S13, and S14).

## AUTHOR CONTRIBUTIONS

**Oleksii Zdorevskyi:** Formal analysis; funding acquisition; supervision. **Erik Endres:** Conceptualization; methodology; data curation; investigation; formal analysis; writing – original draft; visualization; writing – review and editing. **Mai Jousmäki:** Data curation; investigation. **Vivek Sharma:** Conceptualization; supervision; funding acquisition; visualization; project administration; resources; writing – review and editing; methodology; formal analysis. **Volker Zickermann:** Conceptualization; formal analysis. **Cristina Pecorilla:** Investigation; formal analysis. **Mahdi Torabi:** Methodology; data curation; investigation; formal analysis. **Kim Vy Huynh:** Data curation; investigation.

## CONFLICT OF INTEREST STATEMENT

The authors declare no conflicts of interest.

## Supporting information


**Figure S1.** Energetics of charge‐neutral (ε‐nitrogen protonated) histidine sidechain dynamics. The y‐axis in panels (a) and (b) describe the potential of mean force (PMF, kcal/mol) with respect to the reaction coordinate (RC) on x‐axis in Å (see methods). Each trace shown is an average of four simulation replicas, whereas the shaded region describes the standard error of mean. See also Figure 1 in main text. Notations for the protonation states are described in Figure 2 of the main text.
**Figure S2.** Energetics of doubly protonated His254 sidechain conformational dynamics. The y‐axis in panels (a) and (b) describe the potential of mean force (PMF, kcal/mol) with respect to the reaction coordinate (RC) on x‐axis in Å (see methods). Each trace shown is an average of four simulation replicas, whereas the shaded region describes the standard error of mean. See also Figure 1 in the main text.
**Figure S3.** Scatter plot displaying the position of histidine in various 3D structures of complex I and related proteins within the OPM data bank. Proteins that could not be structurally aligned with the membrane‐embedded domain of *E. coli* complex I (PDB 7P7C) or those that did not have histidine present or resolved (17 of 177) were excluded from the plot. See Table S1 for a full list of proteins, and Figure 2 for a depiction of the distances considered.
**Figure S4.** Water and hydrogen bond occupancies in unbiased MD simulations of histidine in A and B conformational states. Simulation snapshots showing hydrogen‐bonded pathways in A position of histidine from two different protonation states (a) 0+0δ and (b) +0+ε. Similarly, panels (c) and (d) display simulation snapshots of histidine in its B conformation from protonation states +++δ and 0+0ε, respectively. Water occupancy is indicated by a gray surface displayed at an iso‐value of 0.5 (meaning that at least for half of the simulation time, a water molecule could be observed in this position). The analysis of the hydrogen bond occupancy was performed with the *WaterBridgeAnalysis* module of *mdanalysis*. Both the water occupancy as well as the hydrogen bond network analysis are based on 3 × 500 ns of unbiased simulation data. For the B conformation of histidine (panels c and d), the underlying simulation data was obtained by extracting a snapshot (from AWH simulations) in B arrangement and performing 3 new unbiased 500 ns simulations for each of the two states (+++δ and 0+0ε). Not all of these replicas stayed in B arrangement; therefore, the data shown in panels (c) and (d) is based on a single replica in each case that stayed consistently in the conformation B of the histidine switch.
**Figure S5.** PMF profiles of proton transfer in A and B conformations of histidine switch. PMF profiles are shown from three independent production simulation replicas (Rep. 1–3, see also methods) for the A position (left panel) and the B position (right panel) of histidine (see Figure 5a,b). The PMF profiles are extracted after one full complete sampling of the reaction coordinate space (see Figure 5 where data from replica # 1 is displayed, and Figure S13 for additional sampling). The time steps of PMF data collection are mentioned in the parenthesis.
**Figure S6.** Time resolved behavior of the reaction coordinate sampling. The dynamics of the reaction coordinate (RC, see Figure S12) is shown for the QM/MM simulations (three replicas, A/B1‐3) in A (upper panel) and B (lower panel) conformation of the histidine switch.
**Figure S7.** Histidine switch substitution in complex I like protein families. Residues in *E. coli* complex I (PDB 7P7C, red cartoon and gray residues) and membrane‐bound Fhl (7Z0S, orange cartoon and cyan residues) occupying similar spatial locations. The membrane‐bound Fhl possesses Ser234 instead of the conserved His254. However, His222 from a neighboring transmembrane helix occupies a similar position as the putative histidine switch. With Lys342/Lys336 and Thr312/Thr292 being conserved as well, Fhl shows the same amino acid residues as the putative proton transfer pathway in RCI.
**Figure S8.** The protein model immersed in lipid bilayer. In this top view from cytoplasmic side, the protein subunits are shown in colored ribbons. The lipids (shades of green, see methods) surrounding the protein are shown with sticks. The Na^+^ and Cl^−^ ions are displayed as blue and orange spheres, respectively. The water solvent is omitted for clarity.
**Figure S9.** Convergence of AWH simulations. The top panels show behavior of four independent AWH simulation replicas for two selected protonation states. Across 24 different simulation setups there are many cases in which the variation in simulation replicas is rather small. We selected these to point out that variation across replicas can be seen in a simulation setup, but overall behavior of PMF profile is consistent across replicas. The lower panels show time dependency of the PMF profile for selected replicas of two simulation setups, highlighting convergence.
**Figure S10.** Equilibrium dynamics of additional degrees of freedom. (a) Histogram of rotation around the Cβ‐Cγ bond of His254 based on 3 × 500 ns of unbiased MD simulation. (b) Time series of the same data displaying multiple back‐and‐forth rotational transitions in every replica. (c) The same dihedral angle based on 4 × 200 ns of biased AWH MD simulations. Similar to unbiased case, both rotamers are populated.
**Figure S11.** pKa values from MD simulation trajectories. For 1.5 μs (3 × 500 ns) of unbiased MD simulations, pKa values of each snapshot were calculated using Propka and plotted, for (a) ++0δ, (b) ++0p, (c) 000δ, (d) +++δ and (e) 0+0δ states. (f) Contact between Glu359 and Lys305 during 3 × 500 ns of unbiased simulations in 0+0δ state. Given the persistence of the contact, we considered the possibility of Glu359 being a proton uptake site and modeled it protonated for the QM/MM simulations (see methods). (g) pKa of Asp178 and Glu144 from selected replica of +++δ state showing that the proton affinities of the two residues are inversely correlated. (H) The overlayed simulation snapshots, corresponding to two frames 200 ns (gray) and 300 ns (cyan), differ only slightly while the pKas of Asp178 and Glu144 vary. Asp178 is protonated in frame 200 ns (Glu144 deprotonated), whereas Glu144 is protonated in frame 300 ns (Asp178 deprotonated). Lys229 and Arg175 are protonated in all snapshots analyzed (see also panel d).
**Figure S12.** Reaction coordinate used in QM/MM simulations. For simulating proton transfer from donor (protonated lysine sidechain) to acceptor (neutral lysine sidechain) via water molecules and polar residues, a reaction coordinate (RC) was sampled with metadynamics approach. RC corresponds to summing the differences of bond distances of hydrogen from donor and acceptor for every hydrogen bond in the pathway. RC = (sum of *red* distances) minus (sum of *blue* distances). The RC values sampled in our QM/MM simulations are [−6.6, +7.2] and [−4.6, +5.3] for A and B cases, respectively (values mentioned are in Å).
**Figure S13.** Convergence of well‐tempered metadynamics‐based QM/MM free energy profiles. PMF profiles with respect to simulation time are shown for three replicas (A/B1‐3) from QM/MM simulations of A (upper panel) and B (lower panel) conformation of histidine. The last 10 ps of data is plotted.
**Figure S14.** Time‐dependent behavior of Gaussian hills height in well‐tempered metadynamics simulations. The data from all three replicas of QM/MM simulations on A and B conformations of histidine are shown. The hill height values corresponding to RC outside the bound range are excluded; due to the simplified nature of the RC, there are some instances when RC goes out of the bound Figure S6 and Figure S12.
**Table S1.** Location of histidine in X‐ray and cryo‐EM structures of RCI and related proteins.
**Table S2.** pK_A_ values calculated by PropKa software for NuoL subunit using PDB structure 7P7C. Amino acids with non‐standard charge assignments at pH = 7 are highlighted. Data for arginine, cysteine and tyrosine residues are not shown here.
**Table S3.** Model systems for classical MD simulations. The table summarizes all combinations of protonation states investigated in this work. All setups were simulated for 3 × 500 ns in an unbiased manner and with AWH for 4 × 200 ns.
**Table S4.** QM/MM setups simulated in this work. Residues marked in red were part of the RC sampled.

**Data S1.** Snapshot from AWH simulation corresponding to histidine in A conformation (see Figure 5a, PDB format).

**Data S2.** Snapshot from AWH simulation corresponding to histidine in B conformation (see Figure 5b, PDB format).

## Data Availability

The data that support the findings of this study are available from the corresponding author upon reasonable request.
