# Supplementary material for: Molecular and energetic basis of histidine switch dynamics in respiratory complex I
Source: Protein Sci. 2026 Jul 12;35(8):e70720. doi: 10.1002/pro.70720 (PMC13358369; doi:10.1002/pro.70720)
Supplement: Supplementary file 1 — Figure S1. Energetics of charge‐neutral (ε‐nitrogen protonated) histidine sidechain dynamics. The y‐axis in panels (a) and (b) describe the potential of mean force (PMF, kcal/mol) with respect to the reaction coordinate (RC) on x‐axis in Å (see methods). Each trace shown is an average of four simulation replicas, whereas the shaded region describes the standard error of mean. See also Figure 1 in main text. Notations for the protonation states are described in Figure 2 of the main text. Figure S2. Energetics of doubly protonated His254 sidechain conformational dynamics. The y‐axis in panels (a) and (b) describe the potential of mean force (PMF, kcal/mol) with respect to the reaction coordinate (RC) on x‐axis in Å (see methods). Each trace shown is an average of four simulation replicas, whereas the shaded region describes the standard error of mean. See also Figure 1 in the main text. Figure S3. Scatter plot displaying the position of histidine in various 3D structures of complex I and related proteins within the OPM data bank. Proteins that could not be structurally aligned with the membrane‐embedded domain of E. coli complex I (PDB 7P7C) or those that did not have histidine present or resolved (17 of 177) were excluded from the plot. See Table S1 for a full list of proteins, and Figure 2 for a depiction of the distances considered. Figure S4. Water and hydrogen bond occupancies in unbiased MD simulations of histidine in A and B conformational states. Simulation snapshots showing hydrogen‐bonded pathways in A position of histidine from two different protonation states (a) 0+0δ and (b) +0+ε. Similarly, panels (c) and (d) display simulation snapshots of histidine in its B conformation from protonation states +++δ and 0+0ε, respectively. Water occupancy is indicated by a gray surface displayed at an iso‐value of 0.5 (meaning that at least for half of the simulation time, a water molecule could be observed in this position). The analysis of the hydrogen bond occupancy [file PRO-35-e70720-s002.docx]

Supplementary file

**Molecular and Energetic Basis of Histidine Switch Dynamics in Respiratory Complex I**

Erik Endres^1^, Mahdi Torabi^1^, Mai Jousmäki^1^, Kim Vy Huynh^1^, Cristina Pecorilla^1^, Oleksii Zdorevskyi^1^, Volker Zickermann^2,3^, Vivek Sharma^1,4^

^1^Department of Physics, University of Helsinki, Helsinki, Finland

^2^Institute of Biochemistry II, University Hospital, Goethe University, 60590 Frankfurt am Main, Germany.

^3^Centre for Biomolecular Magnetic Resonance, Institute for Biophysical Chemistry, Goethe University, 60438 Frankfurt am Main, Germany.

^4^HiLIFE Institute of Biotechnology, University of Helsinki, Helsinki, Finland

Correspondence to;

Vivek Sharma ([vivek.sharma@helsinki.fi](mailto:vivek.sharma@helsinki.fi))

**Fig. S1. Energetics of charge-neutral (ε-nitrogen protonated) histidine sidechain dynamics.** The y-axis in panels (A) and (B) describe the potential of mean force (PMF, kcal/mol) with respect to the reaction coordinate (RC) on x-axis in Å (see methods). Each trace shown is an average of four simulation replicas, whereas the shaded region describes the standard error of mean. See also Fig. 1 in main text. Notations for the protonation states are described in Fig. 2 of the main text.


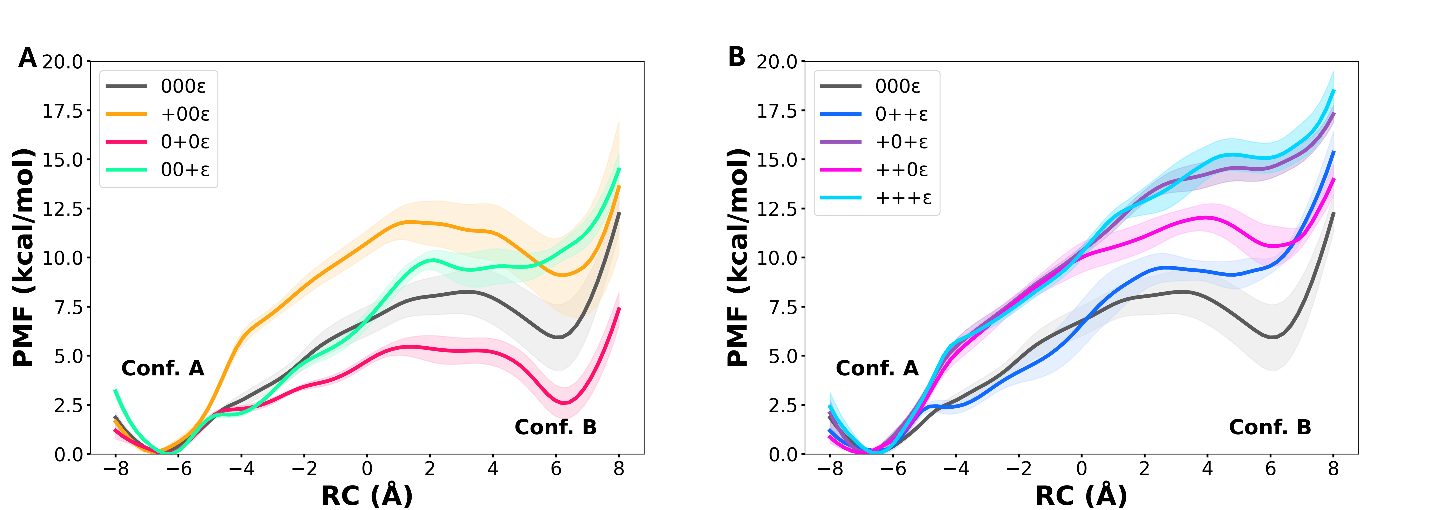


**Fig. S2. Energetics of doubly protonated His254 sidechain conformational dynamics.** The y-axis in panels (A) and (B) describe the potential of mean force (PMF, kcal/mol) with respect to the reaction coordinate (RC) on x-axis in Å (see methods). Each trace shown is an average of four simulation replicas, whereas the shaded region describes the standard error of mean. See also Fig. 1 in the main text.


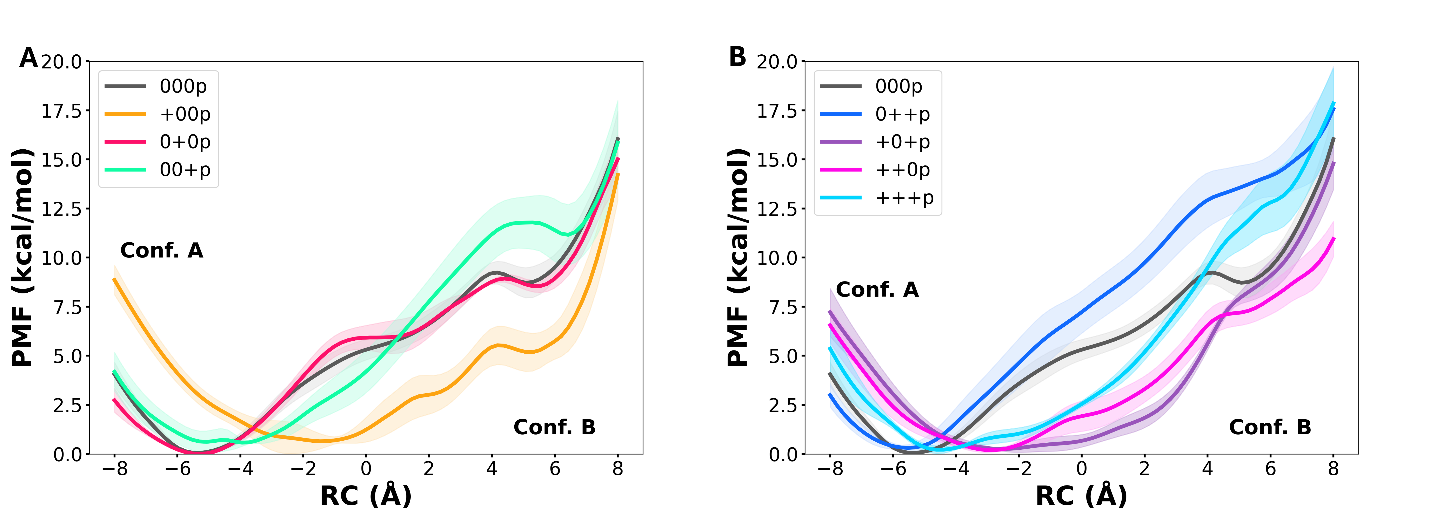


**Fig. S3. Scatter plot displaying the position of histidine in various 3D structures of complex I and related proteins within the OPM data bank**. Proteins that could not be structurally aligned with the membrane-embedded domain of *E. coli* complex I (PDB 7P7C) or those that did not have histidine present or resolved (17 of 177) were excluded from the plot. See Table S1 for a full list of proteins, and Figure 2 for a depiction of the distances considered.


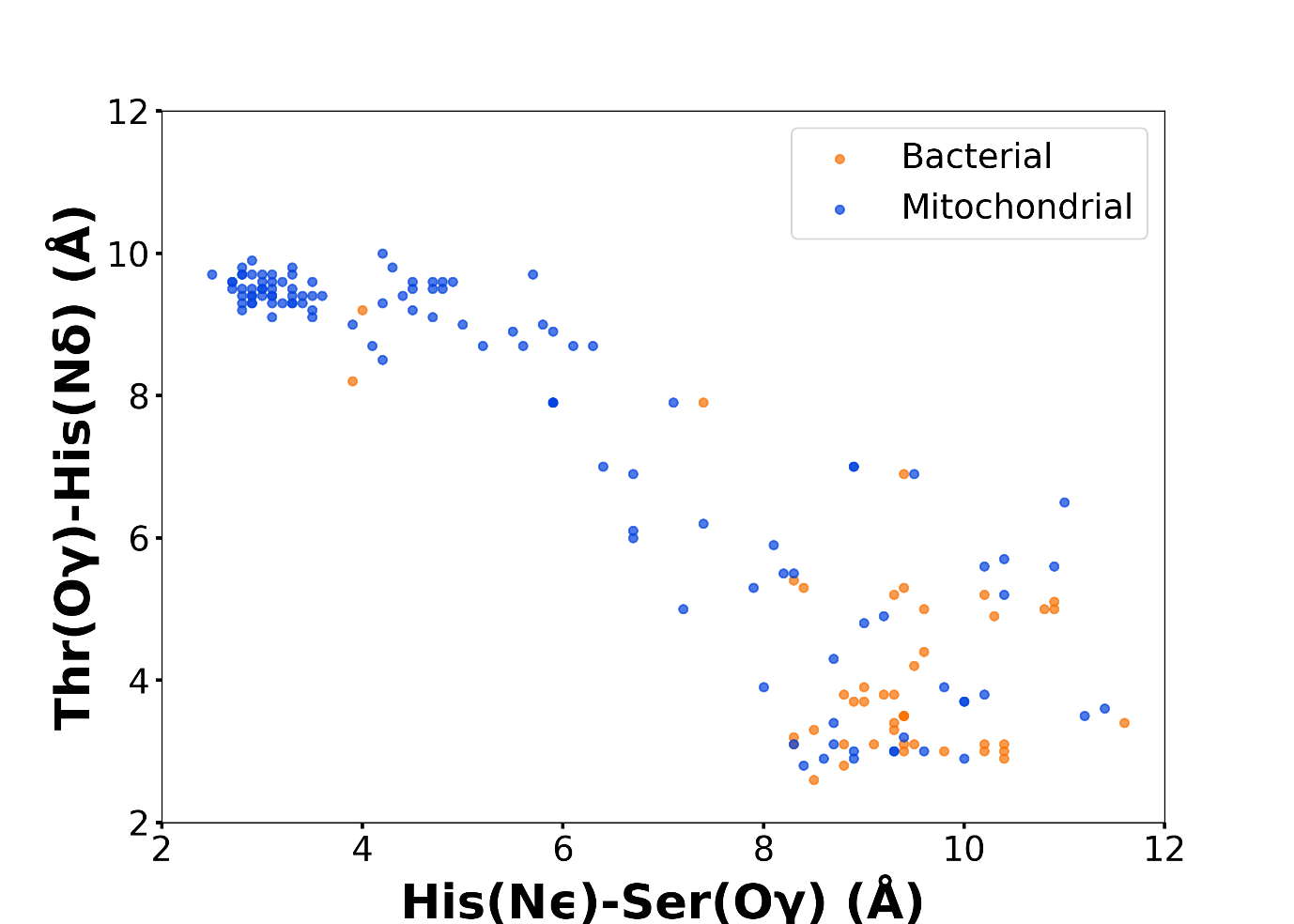


**Fig. S4. Water and hydrogen bond occupancies in unbiased MD simulations of histidine in A and B conformational states.** Simulation snapshots showing hydrogen-bonded pathways in A position of histidine from two different protonation states (A) 0+0δ and (B) +0+ε. Similarly, panels (C) and (D) display simulation snapshots of histidine in its B conformation from protonation states +++δ and 0+0ε, respectively. Water occupancy is indicated by a grey surface displayed at an iso-value of 0.5 (meaning that at least for half of the simulation time, a water molecule could be observed in this position). The analysis of the hydrogen bond occupancy was performed with the *WaterBridgeAnalysis* module of *mdanalysis*. Both the water occupancy as well as the hydrogen bond network analysis are based on 3 x 500 ns of unbiased simulation data. For the B conformation of histidine (panels C and D), the underlying simulation data was obtained by extracting a snapshot (from AWH simulations) in B arrangement and performing 3 new unbiased 500 ns simulations for each of the two states (+++δ and 0+0ε). Not all of these replicas stayed in B arrangement; therefore, the data shown in panels C and D is based on a single replica in each case that stayed consistently in the conformation B of the histidine switch.

**
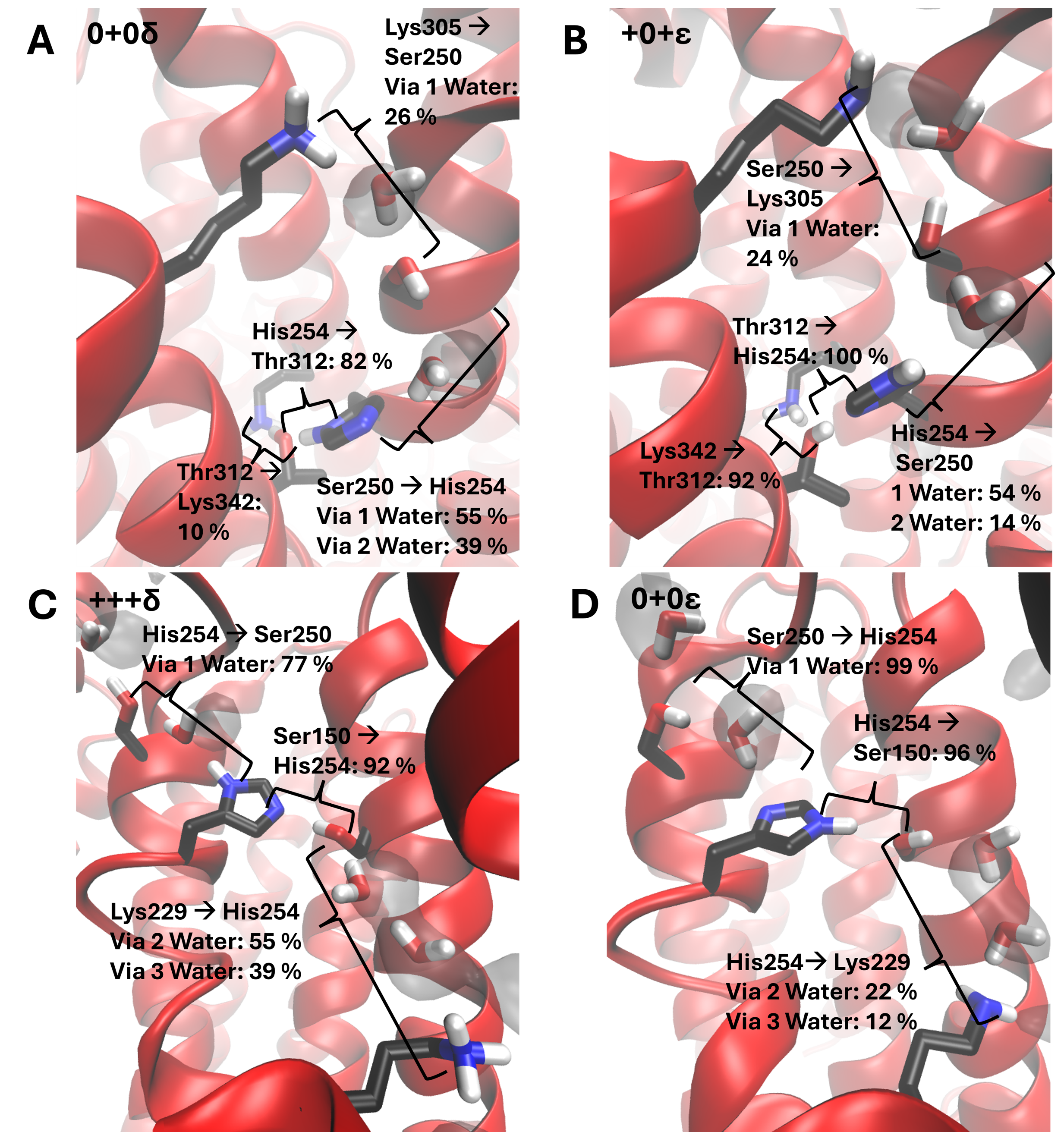
**

**Fig. S5. PMF profiles of proton transfer in A and B conformations of histidine switch.** PMF profiles are shown from three independent production simulation replicas (Rep. 1-3, see also methods) for the A position (left panel) and the B position (right panel) of histidine (see Figs. 5A and 5B). The PMF profiles are extracted after one full complete sampling of the reaction coordinate space (see Fig. 5 where data from replica # 1 is displayed, and Fig. S13 for additional sampling). The time steps of PMF data collection are mentioned in the parenthesis.


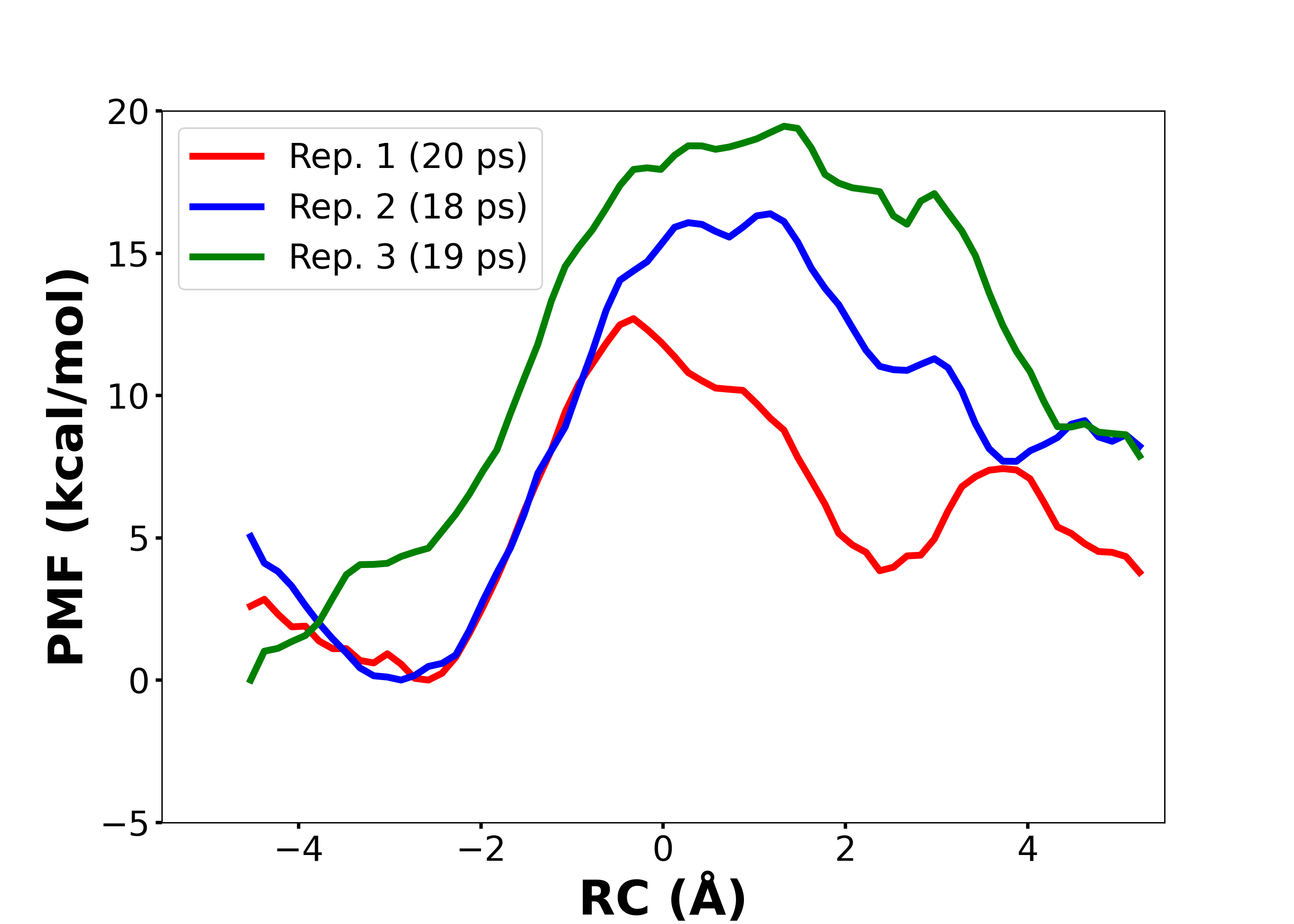


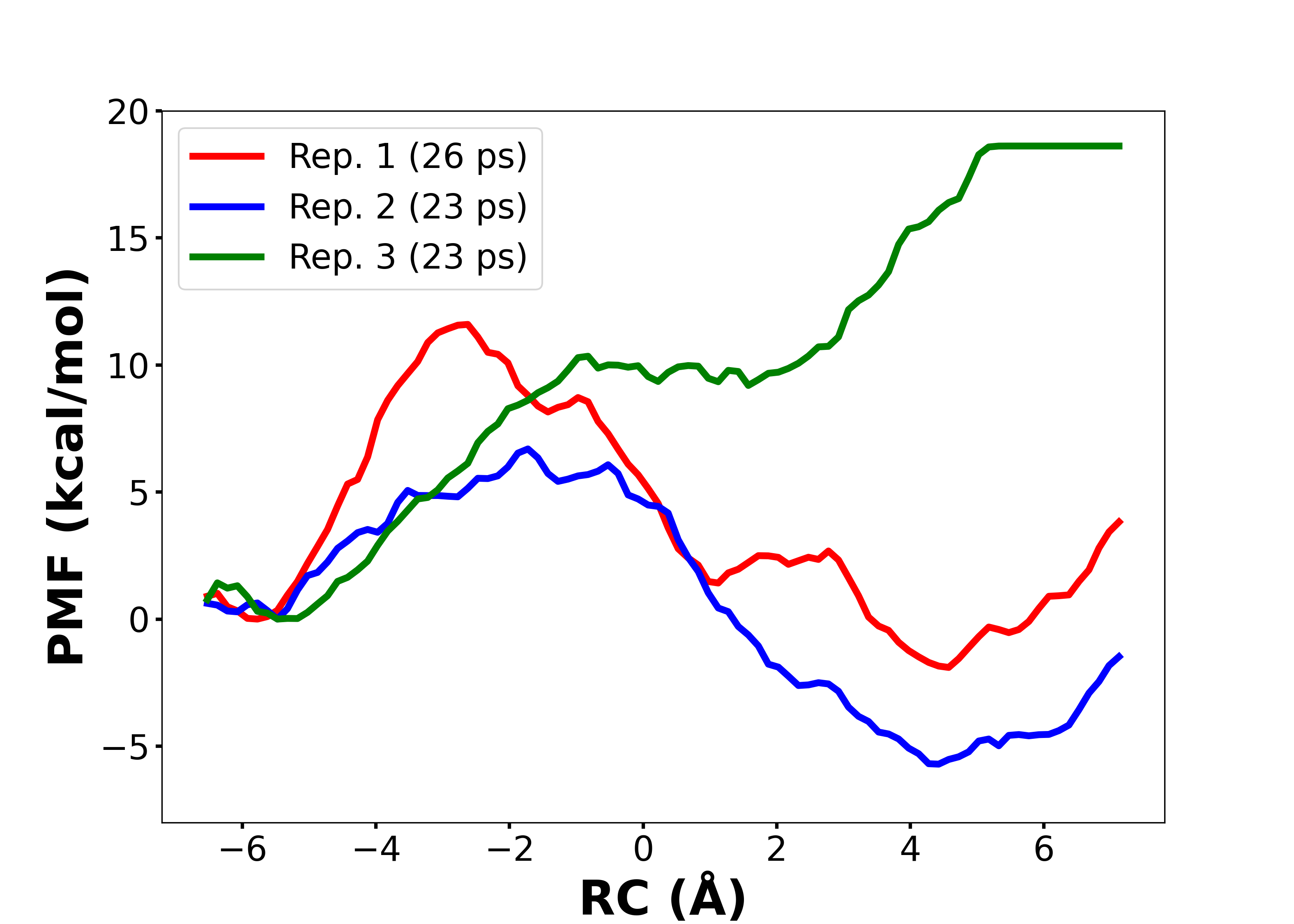


**Fig. S6. Time resolved behaviour of the reaction coordinate sampling.** The dynamics of the reaction coordinate (RC, see Fig. S12) is shown for the QM/MM simulations (three replicas, A/B1-3) in A (upper panel) and B (lower panel) conformation of the histidine switch.


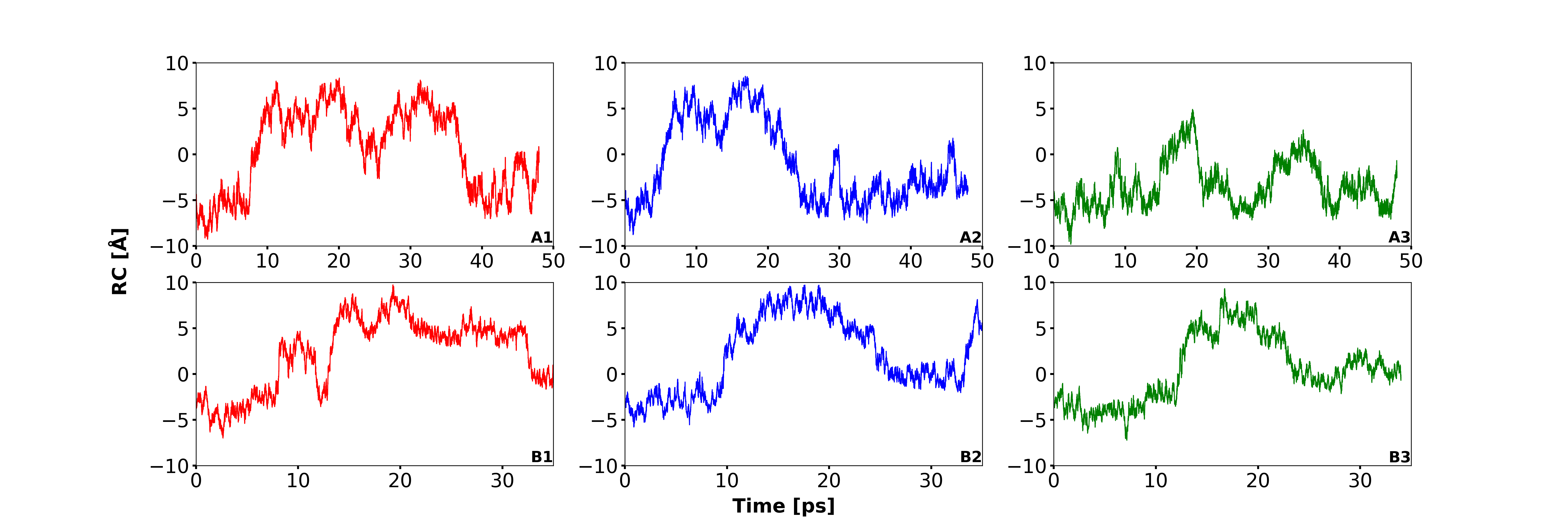


**Fig. S7. Histidine switch substitution in complex I like protein families.** Residues in *E. coli* complex I (PDB 7P7C, red cartoon and grey residues) and membrane-bound Fhl (7Z0S, orange cartoon and cyan residues) occupying similar spatial locations. The membrane-bound Fhl possesses Ser234 instead of the conserved His254. However, His222 from a neighboring transmembrane helix occupies a similar position as the putative histidine switch. With Lys342/Lys336 and Thr312/Thr292 being conserved as well, Fhl shows the same amino acid residues as the putative proton transfer pathway in RCI.


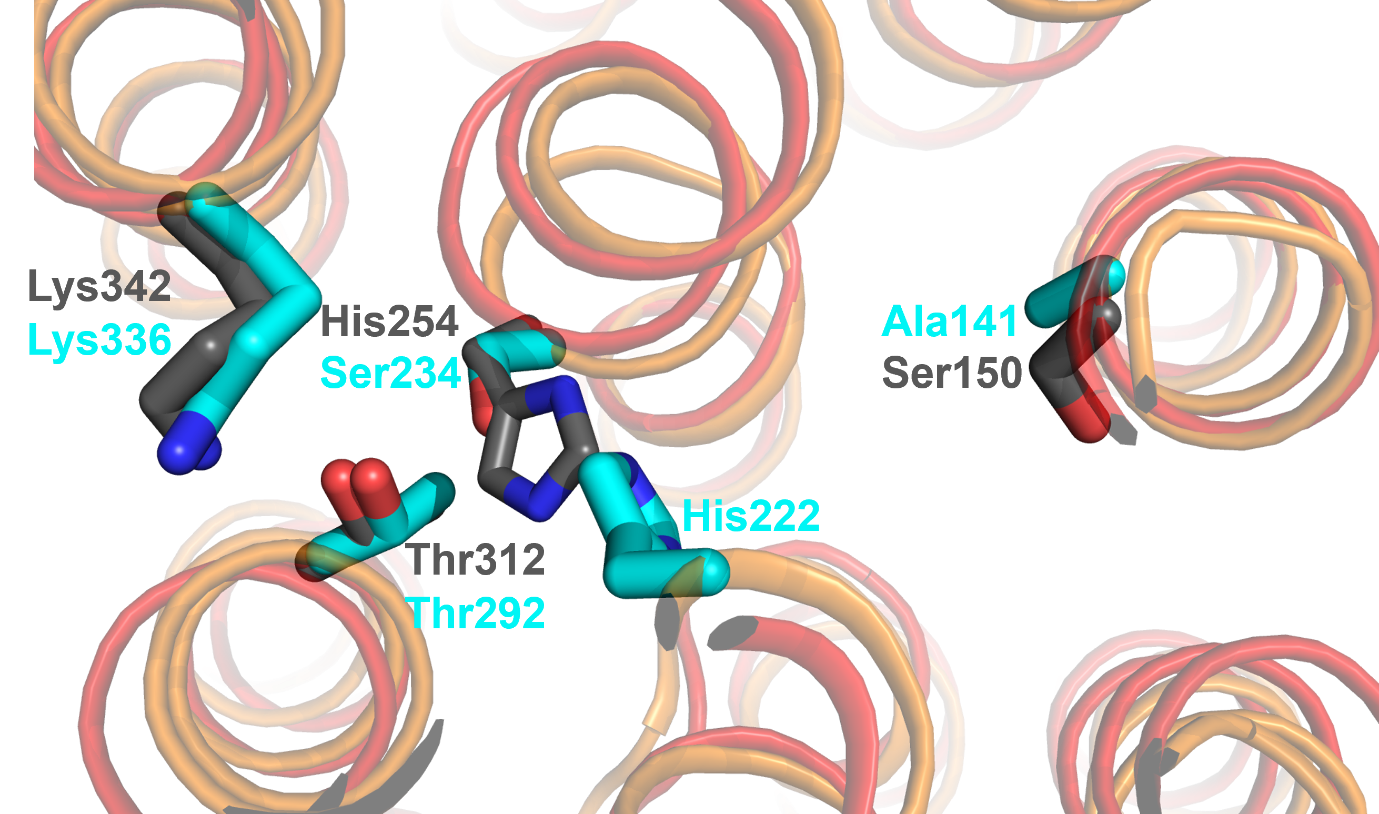


**Fig. S8. The protein model immersed in lipid bilayer.** In this top view from cytoplasmic side, the protein subunits are shown in colored ribbons. The lipids (shades of green, see methods) surrounding the protein are shown with sticks. The Na^+^ and Cl^-^ ions are displayed as blue and orange spheres, respectively. The water solvent is omitted for clarity.


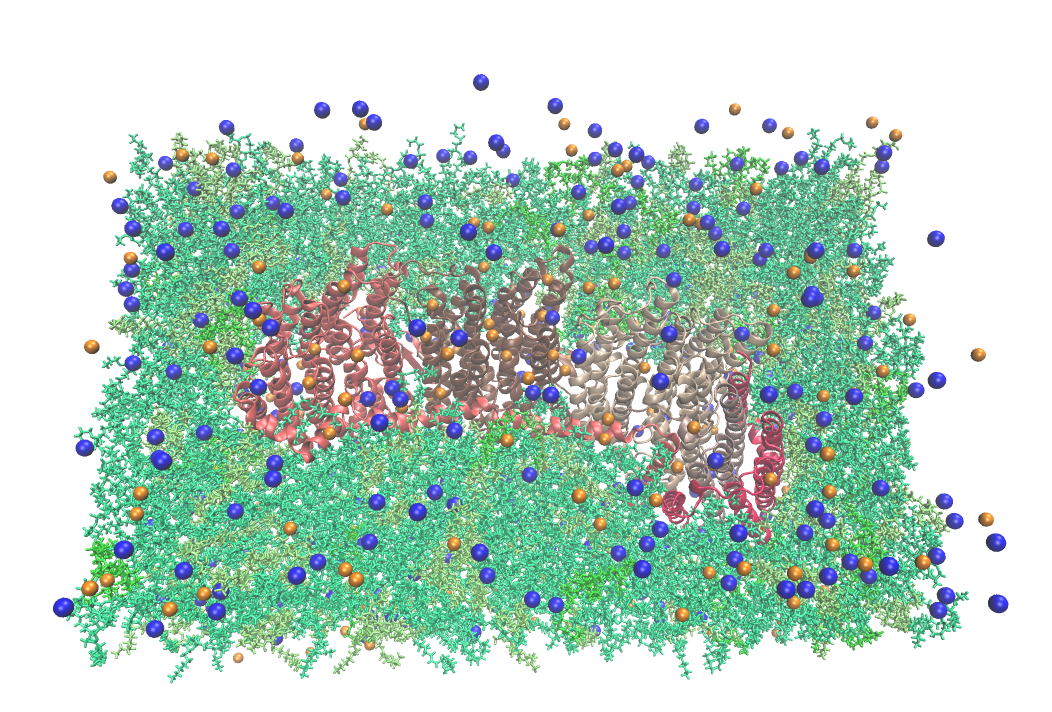


**Fig. S9. Convergence of AWH simulations.** The top panels show behaviour of four independent AWH simulation replicas for two selected protonation states. Across 24 different simulation setups there are many cases in which the variation in simulation replicas is rather small. We selected these to point out that variation across replicas can be seen in a simulation setup, but overall behaviour of PMF profile is consistent across replicas. The lower panels show time dependency of the PMF profile for selected replicas of two simulation setups, highlighting convergence.


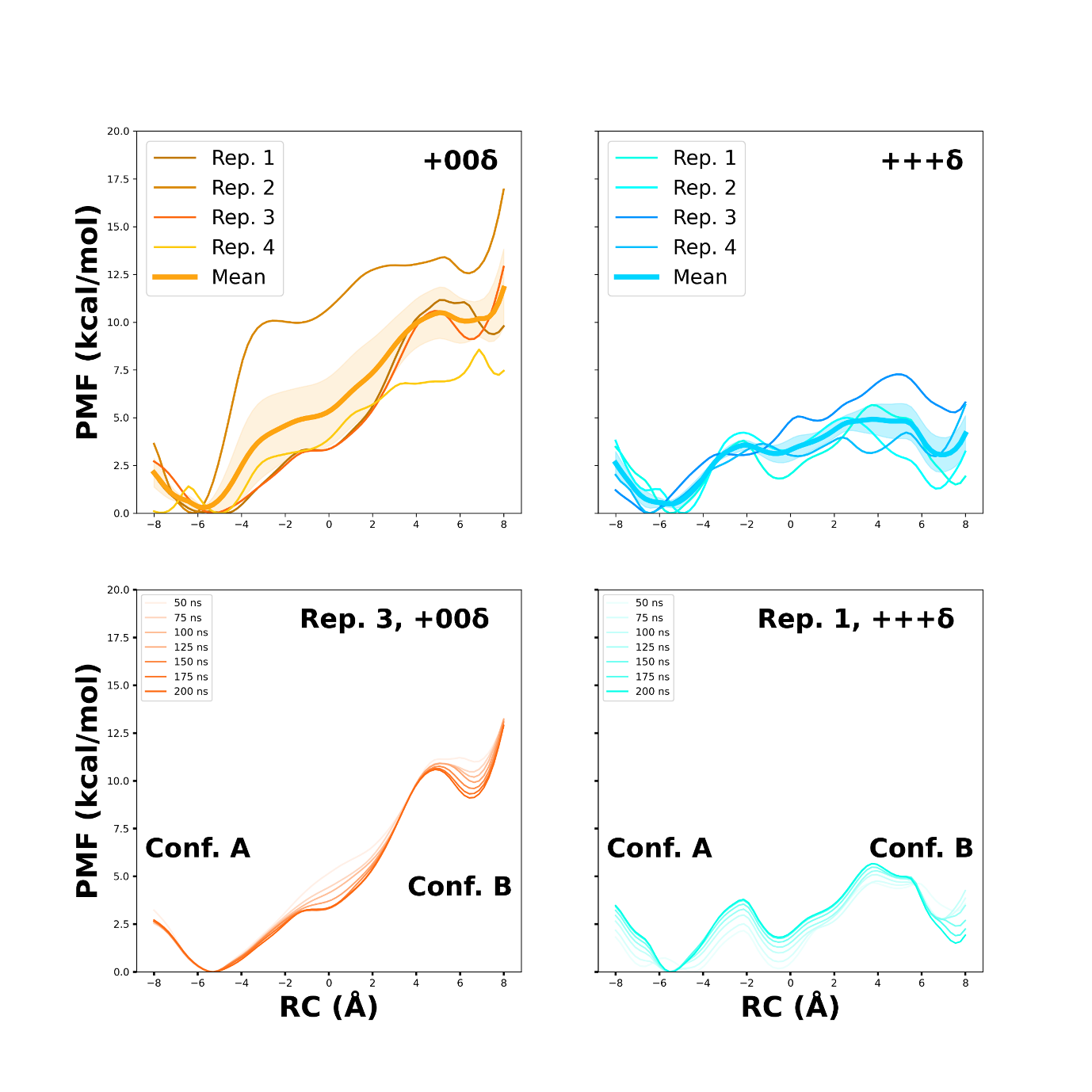


**Fig S10. Equilibrium dynamics of additional degrees of freedom.** (A) Histogram of rotation around the Cβ-Cγ bond of His254 based on 3 x 500 ns of unbiased MD simulation. (B) Time series of the same data displaying multiple back-and-forth rotational transitions in every replica. (C) The same dihedral angle based on 4 x 200 ns of biased AWH MD simulations. Similar to unbiased case, both rotamers are populated.


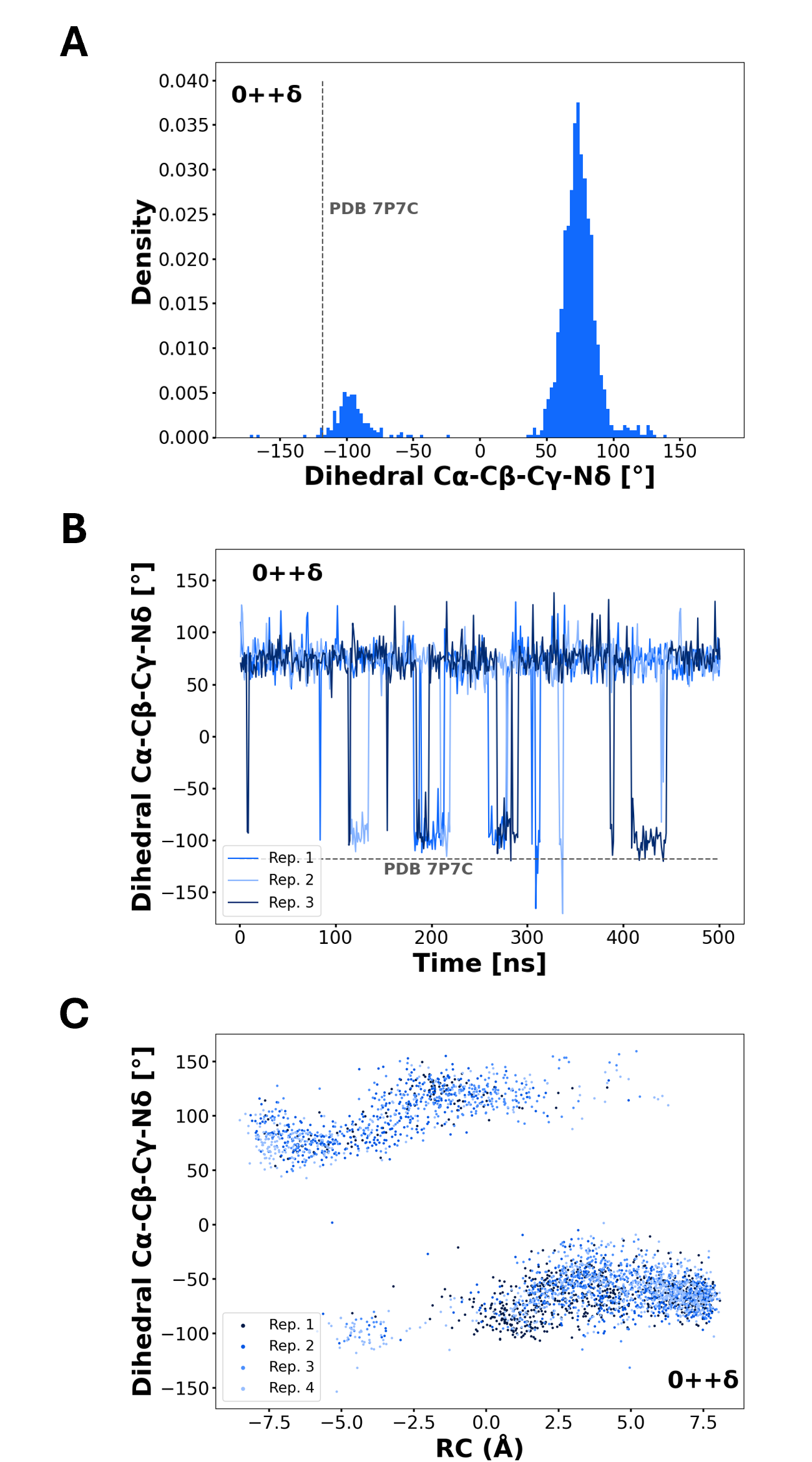


**Fig S11. pKa values from MD simulation trajectories.** For 1.5 μs (3 x 500 ns) of unbiased MD simulations, pka values of each snapshot were calculated using propka and plotted, for (A) ++0δ, (B) ++0p, (C) 000δ, (D) +++δ and (E) 0+0δ states. (F): Contact between Glu359 and Lys305 during 3 x 500 ns of unbiased simulations in 0+0δ state. Given the persistence of the contact, we considered the possibility of Glu359 being a proton uptake site and modelled it protonated for the QM/MM simulations (see methods). (G) pKa of Asp178 and Glu144 from selected replica of +++δ state showing that the proton affinities of the two residues are inversely correlated. (H) The overlayed simulation snapshots, corresponding to two frames 200 ns (grey) and 300 ns (cyan), differ only slightly while the pKas of Asp178 and Glu144 vary. Asp178 is protonated in frame 200 ns (Glu144 deprotonated), whereas Glu144 is protonated in frame 300 ns (Asp178 deprotonated). Lys229 and Arg175 are protonated in all snapshots analyzed (see also panel D).

**
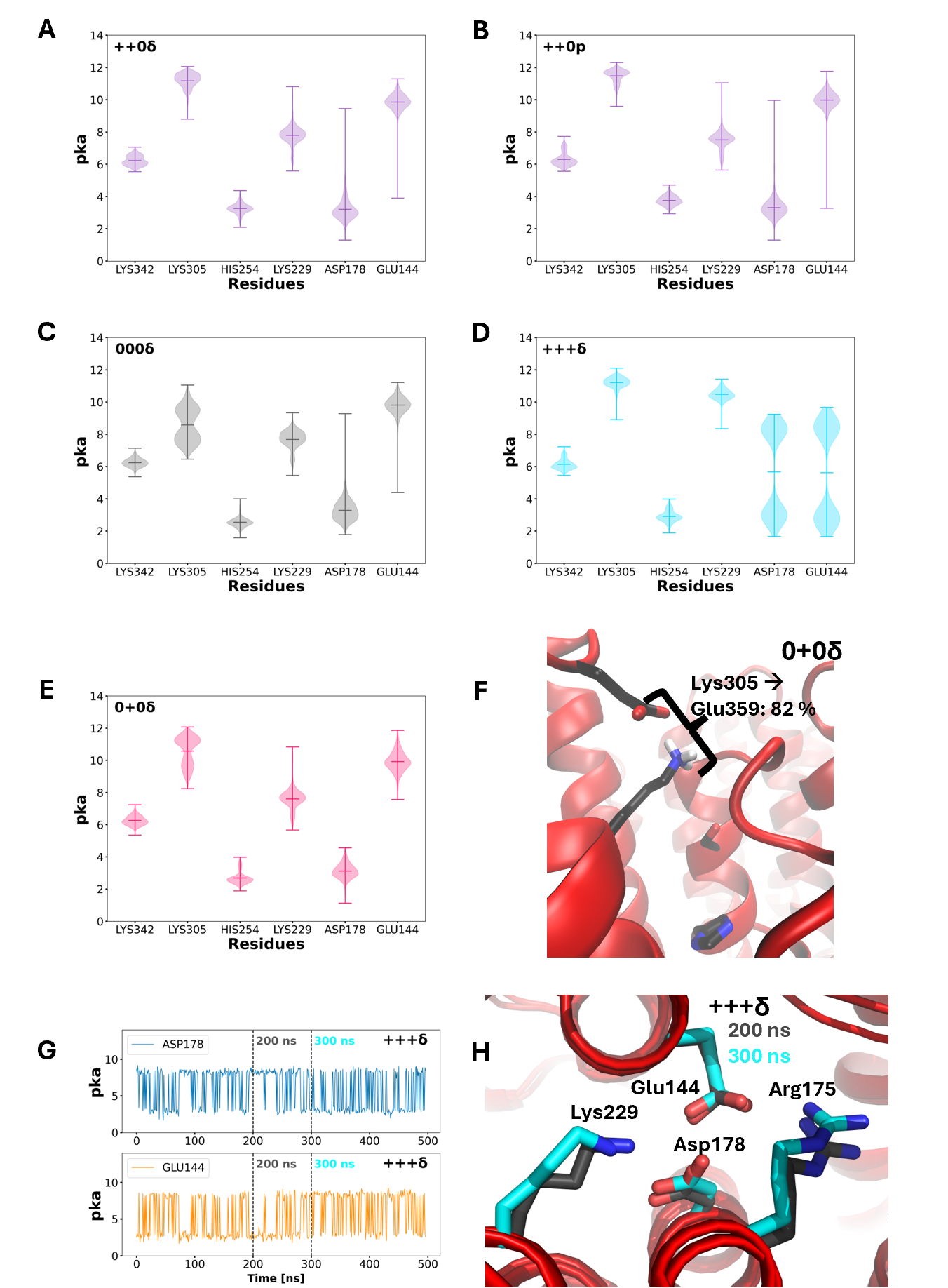
**

**Fig. S12. Reaction coordinate used in QM/MM simulations.** For simulating proton transfer from donor (protonated lysine sidechain) to acceptor (neutral lysine sidechain) via water molecules and polar residues, a reaction coordinate (RC) was sampled with metadynamics approach. RC corresponds to summing the differences of bond distances of hydrogen from donor and acceptor for every hydrogen bond in the pathway. RC = (sum of *red* distances) minus (sum of *blue* distances). The RC values sampled in our QM/MM simulations are [-6.6,+7.2] and [-4.6,+5.3] for A and B cases, respectively (values mentioned are in Å).


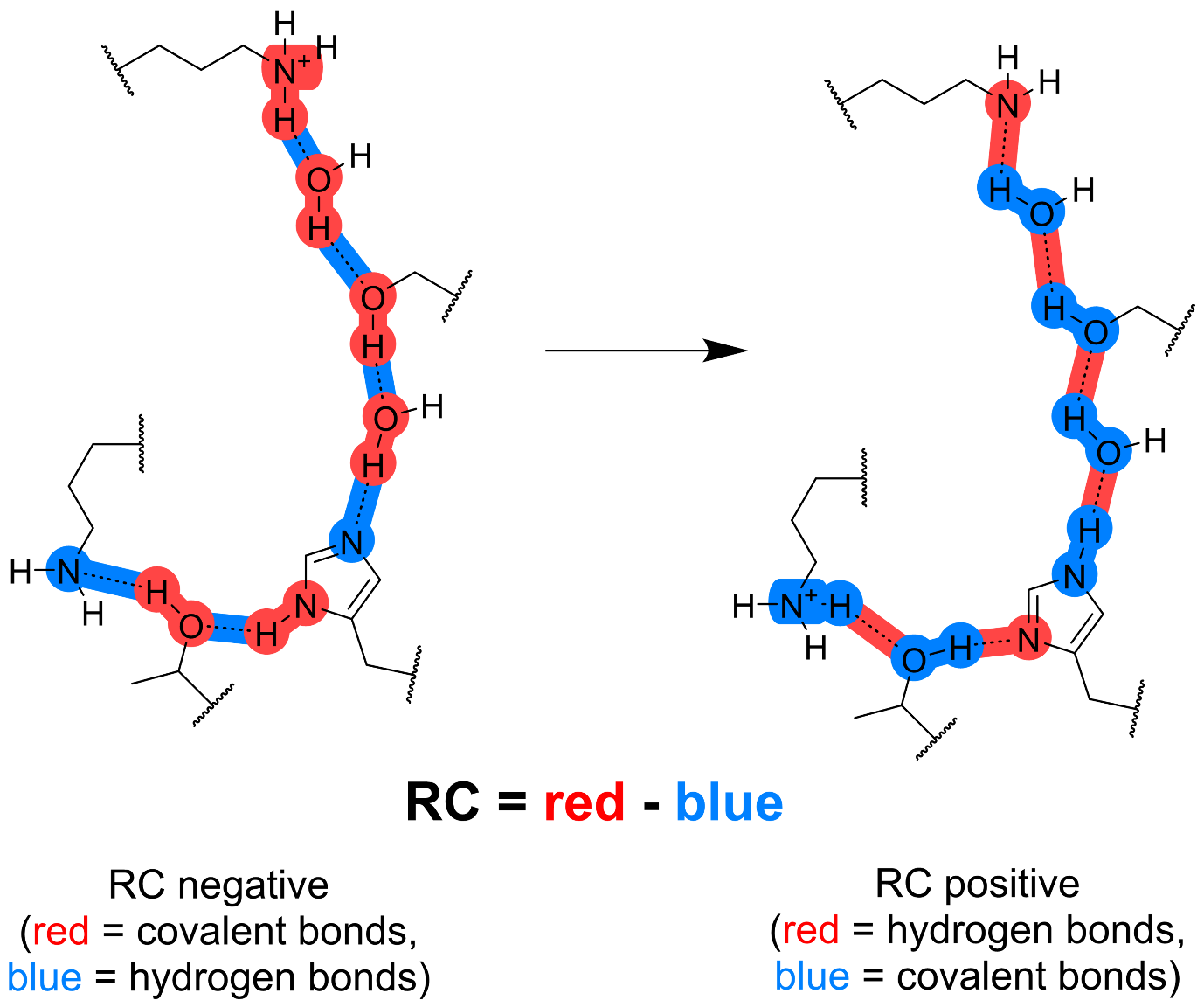


**Fig. S13. Convergence of well-tempered metadynamics-based QM/MM free energy profiles.** PMF profiles with respect to simulation time are shown for three replicas (A/B1-3) from QM/MM simulations of A (upper panel) and B (lower panel) conformation of histidine. The last 10 ps of data is plotted.


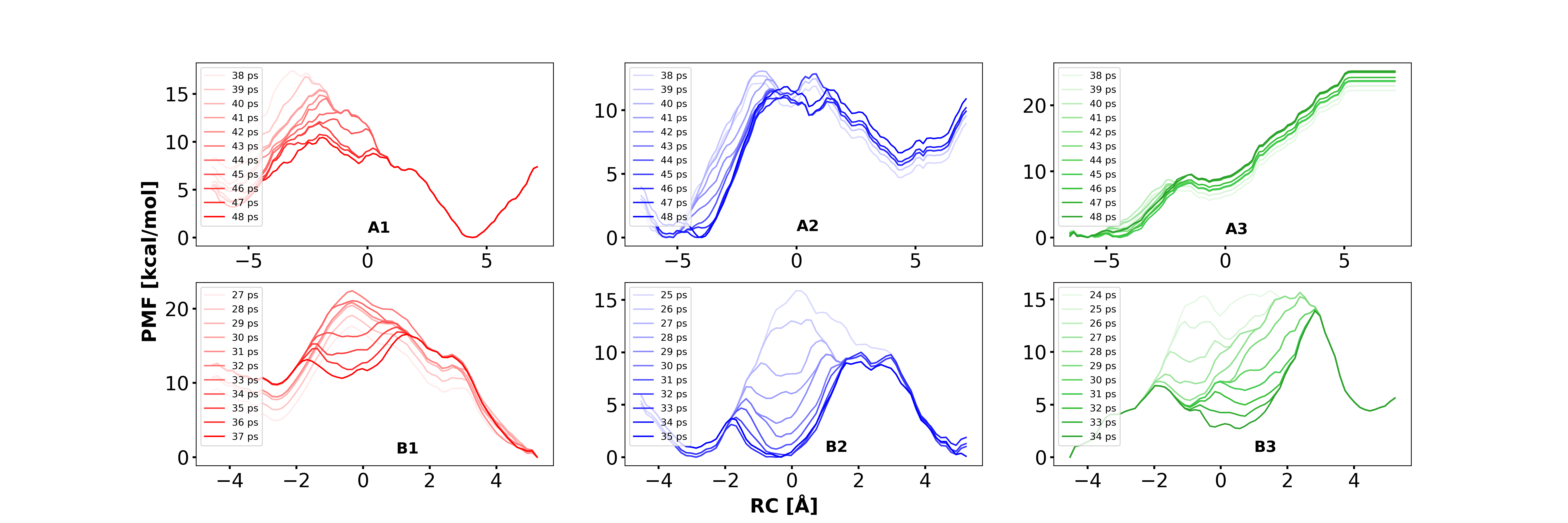


**Fig. S14. Time-dependent behaviour of Gaussian hills height in well-tempered metadynamics simulations.** The data from all three replicas of QM/MM simulations on A and B conformations of histidine are shown. The hill height values corresponding to RC outside the bound range are excluded; due to the simplified nature of the RC, there are some instances when RC goes out of the bound (see Figs. S6 and S12).


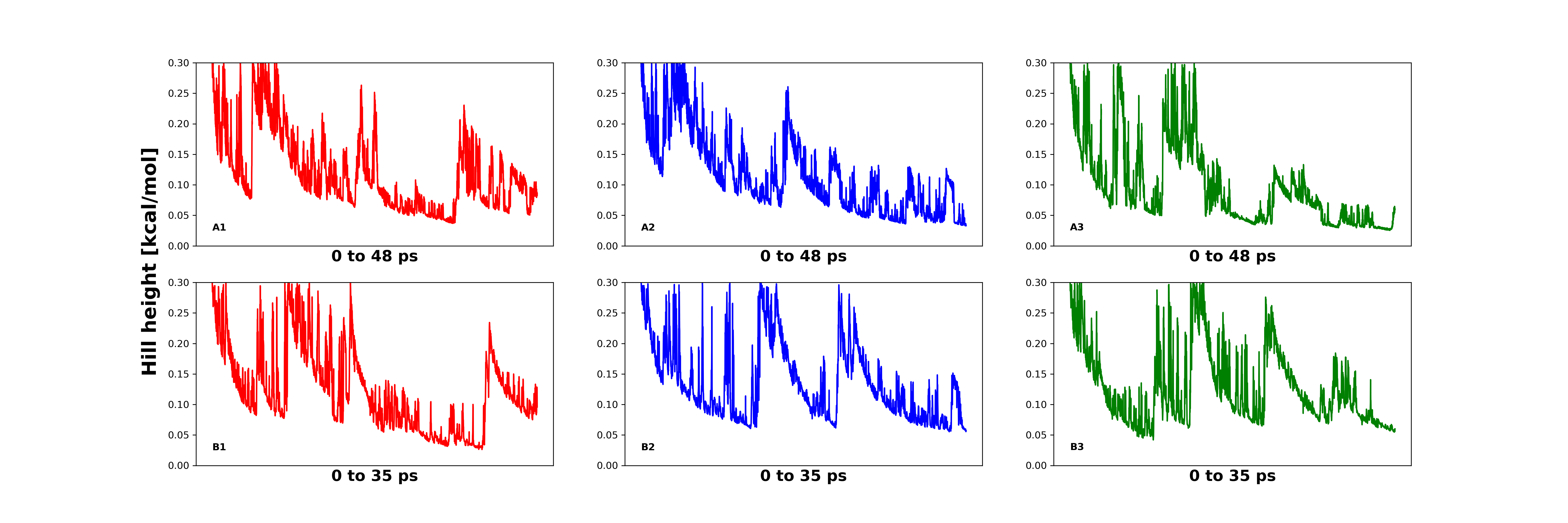


**Table S1. Location of histidine in X-ray and cryo-EM structures of RCI and related proteins.**

| **PDB** | **Resolution (Å)** | **Organism** | **Thr-His(ND1) (Å)** | **Ser-His(NE2) (Å)** | **RC (Å)** |
| --- | --- | --- | --- | --- | --- |
| 7b93 | 3.04 | *Mus musculus* | 9.7 | 5.7 | 4 |
| 7b0n | 3.7 | *Yarrowia lipolytica* | 9.4 | 4.4 | 5 |
| 7v2e | 2.8 | *Sus scrofa* | 9.5 | 4.8 | 4.7 |
| 7ard | 3.11 | *Polytomella sp.* | 3.8 | 10.2 | -6.4 |
| 7qsl | 2.76 | *Bos taurus* | 9.6 | 2.7 | 6.9 |
| 7qsk | 2.84 | *Bos taurus* | 9.7 | 2.5 | 7.2 |
| 8b9z | 3.28 | *Drosophila melanogaster* | 6.9 | 9.5 | -2.6 |
| 7v2h | 2.5 | *Sus scrofa* | 9.5 | 2.8 | 6.7 |
| 7v2c | 2.9 | *Sus scrofa* | 9.7 | 3 | 6.7 |
| 7v2r | 2.6 | *Sus scrofa* | 9.5 | 4.5 | 5 |
| 7v31 | 2.9 | *Sus scrofa* | 9.5 | 2.9 | 6.6 |
| 7v33 | 2.6 | *Sus scrofa* | 9.8 | 3.3 | 6.5 |
| 7z7r | 3.36 | *Escherichia coli* | 2.6 | 8.5 | -5.9 |
| 7p7c | 2.4 | *Escherichia coli* | 5.1 | 10.9 | -5.8 |
| 7p62 | 3.6 | *Escherichia coli* | 4.9 | 10.3 | -5.4 |
| 7p7e | 2.7 | *Escherichia coli* | 2.9 | 10.4 | -7.5 |
| 7zdj | 3.25 | *Ovis aries* | 8.9 | 5.5 | 3.4 |
| 7zeb | 3.8 | *Ovis aries* | 9.9 | 2.9 | 7 |
| 7zdp | 3.88 | *Ovis aries* | 9.3 | 4.2 | 5.1 |
| 7qso | 3.02 | *Bos taurus* | 9.2 | 3.5 | 5.7 |
| 7qsd | 3.1 | *Bos taurus* | 3.9 | 8 | -4.1 |
| 7qsn | 2.81 | *Bos taurus* | 9.4 | 2.9 | 6.5 |
| 7qsm | 2.3 | *Bos taurus* | 9.4 | 2.9 | 6.5 |
| 7v3m | 2.9 | *Sus scrofa* | 9.4 | 2.8 | 6.6 |
| 7v2k | 2.7 | *Sus scrofa* | 9.5 | 4.7 | 4.8 |
| 7v2d | 3.3 | *Sus scrofa* | 9.6 | 4.9 | 4.7 |
| 7v2f | 3.1 | *Sus scrofa* | 10 | 4.2 | 5.8 |
| 7v30 | 2.7 | *Sus scrofa* | 9.6 | 2.7 | 6.9 |
| 7v32 | 3.2 | *Sus scrofa* | 9.6 | 4.8 | 4.8 |
| 6zkc | 3.1 | *Ovis aries* | 9.3 | 3.1 | 6.2 |
| 6zkd | 2.7 | *Ovis aries* | 9.6 | 3.2 | 6.4 |
| 6zke | 2.6 | *Ovis aries* | 9.8 | 2.8 | 7 |
| 6zkf | 2.8 | *Ovis aries* | 9.7 | 2.9 | 6.8 |
| 7zkq | 3.15 | *Yarrowia lipolytica* | complex I assembly intermediate | | |
| 8esw | 3.3 | *Drosophila melanogaster* | 5.7 | 10.4 | -4.7 |
| 7zdm | 3.44 | *Ovis aries* | 9 | 5 | 4 |
| 7zdh | 3.46 | *Ovis aries* | 9.6 | 3.5 | 6.1 |
| 7zd6 | 3.16 | *Ovis aries* | 9.2 | 4.5 | 4.7 |
| 8esz | 3.4 | *Drosophila melanogaster* | 5.3 | 7.9 | -2.6 |
| 7p7m | 3.2 | *Escherichia coli* | 5.2 | 9.3 | -4.1 |
| 6zkk | 3.7 | *Ovis aries* | 9.4 | 3.3 | 6.1 |
| 6zkl | 3.1 | *Ovis aries* | 9.3 | 3.2 | 6.1 |
| 6zkm | 2.8 | *Ovis aries* | 9.7 | 2.8 | 6.9 |
| 6zkn | 2.9 | *Ovis aries* | 9.7 | 2.8 | 6.9 |
| 7zkp | 3.2 | *Yarrowia lipolytica* | complex I assembly intermediate | | |
| 7ar9 | 2.97 | *Polytomella sp.* | 5.6 | 10.9 | -5.3 |
| 7vbl | 2.6 | *Sus scrofa* | 9.3 | 3.3 | 6 |
| 7vc0 | 2.6 | *Sus scrofa* | 9.4 | 3 | 6.4 |
| 7vwl | 2.7 | *Sus scrofa* | 9.5 | 2.7 | 6.8 |
| 7vbp | 2.8 | *Sus scrofa* | 9.6 | 4.7 | 4.9 |
| 8e9h | 2.7 | *Mycolicibacterium smegmatis* | 3.1 | 10.2 | -7.1 |
| 8e9i | 2.8 | *Mycolicibacterium smegmatis* | 5.2 | 10.2 | -5 |
| 8e9g | 2.6 | *Mycolicibacterium smegmatis* | 3 | 10.2 | -7.2 |
| 7o6y | 3.4 | *Yarrowia lipolytica* | 4.8 | 9 | -4.2 |
| 8ba0 | 3.68 | *Drosophila melanogaster* | 5.6 | 10.2 | -4.6 |
| 7z7s | 2.4 | *Escherichia coli* | 3.1 | 8.8 | -5.7 |
| 7p63 | 3.4 | *Escherichia coli* | 3.4 | 11.6 | -8.2 |
| 7z7v | 2.29 | *Escherichia coli* | 2.8 | 8.8 | -6 |
| 7z7t | 3.1 | *Escherichia coli* | 3 | 10.4 | -7.4 |
| 7p64 | 2.5 | *Escherichia coli* | 3.1 | 8.3 | -5.2 |
| 7zci | 2.69 | *Escherichia coli* | 3.1 | 9.5 | -6.4 |
| 7p69 | 3 | *Escherichia coli* | 3.1 | 9.1 | -6 |
| 7z80 | 2.93 | *Escherichia coli* | 3.8 | 8.8 | -5 |
| 7z84 | 2.87 | *Escherichia coli* | 3.2 | 8.3 | -5.1 |
| 7z83 | 2.88 | *Escherichia coli* | 3.3 | 8.5 | -5.2 |
| 7zc5 | 3 | *Escherichia coli* | 3.1 | 9.4 | -6.3 |
| 7p7j | 2.7 | *Escherichia coli* | 5.3 | 9.4 | -4.1 |
| 7p7k | 3.1 | *Escherichia coli* | 5.4 | 8.3 | -2.9 |
| 7p7l | 3 | *Escherichia coli* | 5.3 | 8.4 | -3.1 |
| 6zkg | 3.4 | *Ovis aries* | 9.4 | 3.1 | 6.3 |
| 6zkh | 3 | *Ovis aries* | 9.4 | 3.1 | 6.3 |
| 6zki | 2.8 | *Ovis aries* | 9.6 | 3.1 | 6.5 |
| 6zkj | 3 | *Ovis aries* | 9.4 | 3.6 | 5.8 |
| 7p61 | 3.2 | *Escherichia coli* | 5 | 10.8 | -5.8 |
| 7ak5 | 3.17 | *Mus Musculus* | 8.7 | 6.3 | 2.4 |
| 6zks | 3.1 | *Ovis aries* | 9.7 | 3.3 | 6.4 |
| 6zkt | 2.8 | *Ovis aries* | 9.5 | 3.1 | 6.4 |
| 6zku | 3 | *Ovis aries* | 9.3 | 3.4 | 5.9 |
| 6zkv | 2.9 | *Ovis aries* | 9.4 | 3.5 | 5.9 |
| 7z0t | 3.4 | *Escherichia coli* | Fhl, no histidine | | |
| 7z0s | 2.6 | *Escherichia coli* | Fhl, no histidine | | |
| 3i9v | 3.1 | *Thermus thermophilus* | complex I peripheral arm | | |
| 8bq6 | 2.8 | *Arabidopsis thaliana* | 9.1 | 3.1 | 6 |
| 6zkb | 2.9 | *Ovis aries* | 9.6 | 3 | 6.6 |
| 6zka | 2.5 | *Ovis aries* | 9.7 | 3.1 | 6.6 |
| 6x89 | 3.9 | *Vigna radiata* | His carrying subunit absent | | |
| 7zm7 | 2.77 | *Chaetomium thermophilum* | 9.8 | 4.3 | 5.5 |
| 7zm8 | 2.76 | *Chaetomium thermophilum* | 9.6 | 4.5 | 5.1 |
| 7zmg | 2.44 | *Chaetomium thermophilum* | 9.3 | 2.8 | 6.5 |
| 7zmh | 2.47 | *Chaetomium thermophilum* | 9.2 | 2.8 | 6.4 |
| 7zmb | 2.75 | *Chaetomium thermophilum* | 9 | 3.9 | 5.1 |
| 7zme | 2.83 | *Chaetomium thermophilum* | 9.1 | 4.7 | 4.4 |
| 5xth | 3.9 | *Homo sapiens* | 7.9 | 5.9 | 2 |
| 5gup | 4 | *Sus scrofa* | 8.5 | 4.2 | 4.3 |
| 5xtd | 3.7 | *Homo sapiens* | 7.9 | 5.9 | 2 |
| 5xti | 17.4 | *Homo sapiens* | 7.9 | 5.9 | 2 |
| 6z16 | 2.98 | *Anoxybacillus flavithermus* | 7.9 | 7.4 | 0.5 |
| 7qru | 2.24 | *Alkalihalobacillus pseudofirmus* | 2.8 or 9.3 | 8.8 or 3.0 | -6 or 6.3 |
| 7d3u | 3 | *Dietzia sp.* | 5 | 9.6 | -4.6 |
| 6zko | 3.8 | *Ovis aries* | 9.5 | 3 | 6.5 |
| 6zkp | 3.2 | *Ovis aries* | 9.5 | 3.3 | 6.2 |
| 6zkq | 3.3 | *Ovis aries* | 9.1 | 3.5 | 5.6 |
| 6zkr | 3.5 | *Ovis aries* | 9.4 | 3.4 | 6 |
| 7eu3 | 3.7 | *Hordeum vulgare* | 6.5 | 11 | -4.5 |
| 6zk9 | 2.3 | *Ovis aries* | complex I peripheral arm | | |
| 8bq5 | 2.73 | *Arabidopsis thaliana* | 9.5 | 3 | 6.5 |
| 8bef | 2.13 | *Arabidopsis thaliana* | His carrying subunit absent | | |
| 8bpx | 2.09 | *Brassica oleracea* | 9.3 | 2.9 | 6.4 |
| 8beh | 2.29 | *Brassica oleracea* | 9.3 | 2.9 | 6.4 |
| 7a23 | 3.7 | *Brassica oleracea* | 5.2 | 10.4 | -5.2 |
| 7a24 | 3.8 | *Brassica oleracea* | complex I assembly intermediate | | |
| 7ar8 | 3.53 | *Brassica oleracea* | 7.9 | 7.1 | 0.8 |
| 7arb | 3.41 | *Brassica oleracea* | 8.7 | 5.2 | 3.5 |
| 7aqq | 3.06 | *Brassica oleracea* | His carrying subunit absent | | |
| 7aqw | 3.17 | *Brassica oleracea* | 8.7 | 6.1 | 2.6 |
| 7ar7 | 3.72 | *Brassica oleracea* | 9 | 5.8 | 3.2 |
| 7nyh | 3.6 | *Escherichia coli* | 3.5 | 9.4 | -5.9 |
| 2ybb | 19 | *Bos taurus* | Sidechain not modeled | | |
| 7nyr | 3.3 | *Escherichia coli* | 3.5 | 9.4 | -5.9 |
| 6cfw | 3.7 | *Pyrococcus furiosus* | Mbh, histidine likely not conserved | | |
| 3rko | 3 | *Escherichia coli* | 5 | 10.9 | -5.9 |
| 7ak6 | 3.82 | *Mus musculus* | 8.7 | 4.1 | 4.6 |
| 7nyv | 3.7 | *Escherichia coli* | 3.5 | 9.4 | -5.9 |
| 7nyu | 3.8 | *Escherichia coli* | 3.5 | 9.4 | -5.9 |
| 6zr2 | 3.1 | *Mus musculus* | 8.7 | 5.6 | 3.1 |
| 6u8y | 4 | *Pyrococcus furiosus* | Mbs, histidine likely not conserved | | |
| 5ldw | 4.27 | *Bos taurus* | 3.7 | 10 | -6.3 |
| 5lc5 | 4.35 | *Bos taurus* | 3.9 | 9.8 | -5.9 |
| 5ldx | 5.6 | *Bos taurus* | 3.7 | 10 | -6.3 |
| 7dgz | 3.8 | *Bos taurus* | 6.1 | 6.7 | -0.6 |
| 6ztq | 3 | *Mus musculus* | 8.9 | 5.9 | 3 |
| 5o31 | 4.13 | *Bos taurus* | 4.3 | 8.7 | -4.4 |
| 6hum | 3.34 | *Thermosynechococcus elongatus* | 6.9 | 9.4 | -2.5 |
| 6nby | 3.1 | *Thermosynechococcus elongatus* | 3.9 | 9 | -5.1 |
| 6nbq | 3.1 | *Thermosynechococcus elongatus* | Sidechain not resolved | | |
| 6nbx | 3.5 | *Thermosynechococcus elongatus* | 3.8 | 9.2 | -5.4 |
| 6khi | 3 | *Thermosynechococcus elongatus* | 8.2 | 3.9 | 4.3 |
| 6khj | 3 | *Thermosynechococcus elongatus* | 9.2 | 4 | 5.2 |
| 6l7o | 3.2 | *Thermosynechococcus elongatus* | 4.2 | 9.5 | -5.3 |
| 6l7p | 3.6 | *Thermosynechococcus elongatus* | 4.4 | 9.6 | -5.2 |
| 6tjv | 3.2 | *Thermosynechococcus elongatus* | Arginine instead of histidine | | |
| 4wz7 | 3.6 | *Yarrowia lipolytica* | 3.5 | 11.2 | -7.7 |
| 6rfr | 3.2 | *Yarrowia lipolytica* | 5.5 | 8.3 | -2.8 |
| 6h8k | 3.79 | *Yarrowia lipolytica* | 3.6 | 11.4 | -7.8 |
| 6rfq | 3.3 | *Yarrowia lipolytica* | 5.5 | 8.2 | -2.7 |
| 6yj4 | 2.7 | *Yarrowia lipolytica* | 9.3 | 3.3 | 6 |
| 7dh0 | 4.2 | *Bos taurus* | 7 | 8.9 | -1.9 |
| 5lnk | 3.9 | *Ovis aries* | 4.9 | 9.2 | -4.3 |
| 4hea | 3.3 | *Thermus thermophilus* | 3 | 9.4 | -6.4 |
| 6y11 | 3.11 | *Thermus thermophilus* | 3.7 | 8.9 | -5.2 |
| 6i1p | 3.21 | *Thermus thermophilus* | 3.3 | 9.3 | -6 |
| 6i0d | 3.6 | *Thermus thermophilus* | 3 | 9.8 | -6.8 |
| 6q8o | 3.61 | *Thermus thermophilus* | 3.7 | 9 | -5.3 |
| 6q8w | 3.4 | *Thermus thermophilus* | 3.4 | 9.3 | -5.9 |
| 6q8x | 3.51 | *Thermus thermophilus* | 3.8 | 9.3 | -5.5 |
| 6ziy | 4.25 | *Thermus thermophilus* | 3.1 | 10.4 | -7.3 |
| 6zjy | 5.5 | *Thermus thermophilus* | Sidechain not resolved | | |
| 5xtc | 3.7 | *Homo sapiens* | 7.9 | 5.9 | 2 |
| 6qc6 | 4.1 | *Ovis aries* | 6.2 | 7.4 | -1.2 |
| 7r41 | 2.3 | *Bos taurus* | 3.1 | 8.3 | -5.2 |
| 7r42 | 2.3 | *Bos taurus* | 2.8 | 8.4 | -5.6 |
| 7r43 | 2.4 | *Bos taurus* | 3.1 | 8.7 | -5.6 |
| 7r44 | 2.4 | *Bos taurus* | 2.9 | 8.6 | -5.7 |
| 7r45 | 2.4 | *Bos taurus* | 3 | 9.6 | -6.6 |
| 7r46 | 2.4 | *Bos taurus* | 3.4 | 8.7 | -5.3 |
| 7r47 | 2.3 | *Bos taurus* | 3 | 8.9 | -5.9 |
| 7r48 | 2.3 | *Bos taurus* | 3.2 | 9.4 | -6.2 |
| 7r4c | 2.3 | *Bos taurus* | 3 | 9.3 | -6.3 |
| 7r4d | 2.3 | *Bos taurus* | 2.9 | 10 | -7.1 |
| 7r4f | 2.4 | *Bos taurus* | 3 | 9.3 | -6.3 |
| 7r4g | 2.5 | *Bos taurus* | 2.9 | 8.9 | -6 |
| 6q9b | 3.9 | *Ovis aries* | 7 | 6.4 | 0.6 |
| 6qa9 | 4.1 | *Ovis aries* | 6.9 | 6.7 | 0.2 |
| 6qc4 | 4.6 | *Ovis aries* | 5 | 7.2 | -2.2 |
| 7dgr | 4.6 | *Bos taurus* | 5.9 | 8.1 | -2.2 |
| 7dgs | 7.8 | *Bos taurus* | 6 | 6.7 | -0.7 |
| 7dkf | 8.3 | *Bos taurus* | 7 | 8.9 | -1.9 |

**Table S2. pK_A_ – values calculated by PropKa software for NuoL subunit using PDB structure 7P7C.** Amino acids with non-standard charge assignments at pH = 7 are highlighted. Data for arginine, cysteine and tyrosine residues are not shown here.

| ***Residue*** | ***pka*** | ***State*** | ***Residue*** | ***pka*** | ***State*** |
| --- | --- | --- | --- | --- | --- |
| Asp51 | 3.93 | Negative | His100 | 3.84 | Neutral |
| Asp72 | 3.94 | Negative | His207 | 7.16 | Positive |
| Asp82 | 2.07 | Negative | His254 | 2.81 | Neutral |
| Asp134 | 5.74 | Negative | His270 | 4.98 | Neutral |
| Asp160 | 3.65 | Negative | His281 | 5.39 | Neutral |
| Asp178 | 4.81 | Negative | His334 | 2.39 | Neutral |
| Asp210 | 3.89 | Negative | His338 | 0.10 | Neutral |
| Asp241 | 3.14 | Negative | His357 | 6.44 | Neutral |
| Asp303 | 4.53 | Negative | His358 | 6.74 | Neutral |
| Asp329 | 6.10 | Negative | His411 | 5.84 | Neutral |
| Asp400 | 7.28 | Neutral | His438 | 5.39 | Neutral |
| Asp542 | 5.65 | Negative | His487 | 6.43 | Neutral |
| Asp546 | 3.95 | Negative | Lys162 | 10.58 | Positive |
| Asp563 | 1.65 | Negative | Lys169 | 12.02 | Positive |
| Glu27 | 3.98 | Negative | Lys229 | 9.90 | Positive |
| Glu57 | 4.91 | Negative | Lys305 | 7.80 | Positive |
| Glu110 | 4.91 | Negative | Lys342 | 5.99 | Neutral |
| Glu111 | 3.62 | Negative | LYS364 | 10.53 | Positive |
| Glu144 | 9.92 | Neutral | LYS370 | 10.20 | Positive |
| Glu191 | 4.24 | Negative | LYS399 | 7.17 | Positive |
| Glu202 | 4.61 | Negative | LYS515 | 10.41 | Positive |
| Glu278 | 4.63 | Negative | LYS547 | 10.35 | Positive |
| Glu401 | 6.32 | Negative | LYS551 | 10.50 | Positive |
| Glu484 | 4.68 | Negative | LYS561 | 9.96 | Positive |
| Glu494 | 4.51 | Negative | LYS581 | 10.13 | Positive |
| Glu587 | 9.56 | Neutral |  |  |  |

**Table S3. Model systems for classical MD simulations.** The table summarizes all combinations of protonation states investigated in this work. All setups were simulated for 3 x 500 ns in an unbiased manner and with AWH for 4 x 200 ns.

| **Lys342** | **Lys305** | **Lys229** | **His254** |
| --- | --- | --- | --- |
| 0 | + | + | δ |
| 0 | + | + | ε |
| 0 | + | + | p |
| 0 | 0 | + | δ |
| 0 | 0 | + | ε |
| 0 | 0 | + | p |
| + | + | + | δ |
| + | + | + | ε |
| + | + | + | p |
| 0 | + | 0 | δ |
| 0 | + | 0 | ε |
| 0 | + | 0 | p |
| 0 | 0 | 0 | δ |
| 0 | 0 | 0 | ε |
| 0 | 0 | 0 | p |
| + | + | 0 | δ |
| + | + | 0 | ε |
| + | + | 0 | p |
| + | 0 | 0 | δ |
| + | 0 | 0 | ε |
| + | 0 | 0 | p |
| + | 0 | + | δ |
| + | 0 | + | ε |
| + | 0 | + | p |

**Table S4. QM/MM setups simulated in this work.** Residues marked in red were part of the RC sampled.

| **Conformational state of histidine and snapshot origin** | **Protonation state simulated for three lysine residues and histidine (other residues)** | **Number of QM atoms** | **Number of MM atoms** | **Residues in QM-Region** | **Total charge of QM-Region** | **Simulation time (~ps)** |
| --- | --- | --- | --- | --- | --- | --- |
| A (snapshot from AWH simulation of 0+0δ state) | 0+0δ (all other residues in propka-predicted protonation states, except Glu359 modeled neutral) | 168 | 310246 | L239, M243, S250, I253, H254(δ), K305(+), S311, T312, K342(0), H338(δ), F346,  E359(0), 10+2 water molecules | +1 | 3 replicas x 45 ps |
| B (snapshot from AWH simulation of +++δ state) | +++δ (all other residues in propka-predicted protonation states, except Asp178 modeled neutral) | 172 | 310248 | W143, E144(0), S150, I154, F171, T174, R175(+), D178(0), K229(+), W238, S250, H254(δ), M258, 4+2 water molecules | +2 | 3 replicas x 35 ps |
| A (snapshot from AWH simulation of 0+0δ state, see supporting file 1) | 0+0δ (all other residues in propka-predicted protonation states) | 167 | 310245 | L239, M243, S250, I253, H254(δ), K305(+), S311, T312, K342(0), H338(δ), F346,  E359(0), 10+2 water molecules | 0 | 1 replica x 30 ps |
| B (snapshot from AWH simulation of +++δ state, see supporting file 2) | +++δ (all other residues in propka-predicted protonation states) | 171 | 310247 | W143, E144(0), S150, I154, F171, T174, R175(+), D178(0), K229(+), W238, S250, H254(δ), M258, 4+2 water molecules | +1 | 1 replica x 20 ps |

**Supplementary note**

**Mechanistic aspects**

The redox-coupled proton pumping mechanism of respiratory complex I remains unknown and is extensively discussed in the field (Sazanov 2023; Djurabekova et al. 2024; Kaila 2018). Based on the MD simulations of a 3.3 Å X-ray structure of *T. thermophilus* complex I, we discovered the protonation-state dependent dynamics of conserved His254 (Djurabekova, Haapanen, and Sharma 2020). The high-resolution cryo-EM structure (2.2 Å) of evolutionarily related Mrp antiporter provided direct evidence of the two states of histidine residue (called conformations A and B here (Lee et al. 2022). Further site-directed mutagenesis studies on genetically accessible Mrp-antiporter and MD simulations of WT and mutant protein confirmed the importance of histidine and its conformational dynamics in protein function (Pecorilla et al. 2025). In the histidine switch mechanism described by Pecorilla et al. (Fig. S29 in Pecorilla et al. 2025), a proton is picked up from the N side of the membrane involving Lys305 and is transferred to the buried (and initially proton deficient) Lys342 via His254, which is stabilized in the A conformation. Our current AWH MD simulations and QM/MM calculations provide support to the proposed model. Importantly, histidine stabilized in the A conformation shuts off the connectivity to the central hydrophilic axis, thereby effectively preventing any proton transfer from the N side via NuoL subunit to the quinone site. This gating by histidine switch would be especially important in states when quinone-reduction derived negative charges are built-up in the system to drive the pump; premature protonation (neutralization) of negative charges, in the absence of any gating, can lead to wasteful loss of energy.

The trigger for histidine switch to move from state A to state B comes from the protonation of KE-pair region. Indeed, in our free energy calculations of +++δ state, the energy of A conformational state comes at par with the energy of the B state upon protonation of Lys229, therefore could lead to the switching of histidine from position A to position B, as postulated previously (Pecorilla et al. 2025). The origin of proton on Lys229 remains unknown, but protonation of Lys229 could be triggered by the quinone reduction reaction at the redox active site, as has been suggested based on the proton-injection driven proton pumping mechanism (Parey et al. 2021; Djurabekova et al. 2024). Importantly, stabilization of histidine in B position disconnects the P-side proton path of NuoL subunit with the N-side (Lys305) path, preventing the loss of proton loaded on Lys342 (or in the region) to the cytoplasmic side. Next, the protonation of His254 (in B position) from Lys229, which is also observed in our QM/MM simulations, is helped by the transient protonation of Asp178. This highly conserved acidic residue is part of the charged cluster (Lys229, Glu144, Arg175, Asp178) located at the interface of NuoL and NuoM subunits of complex I and may participate in transfer of protons from the central hydrophilic axis to Lys229 as part of the quinone redox chemistry (see also Parey et al. 2021; Pecorilla et al. 2025). The proton transfer from Lys229 to His254 triggers the departure of protonated histidine from state B to an intermediate state in agreement with the previous proposal (Pecorilla et al. 2025). This unique conformation and protonated state of histidine can drive the pumping of proton loaded on Lys342 (or in the region) to the P side of the membrane due to electrostatic repulsion. The current model does not take into account the additional gates that may function to prevent the loss of proton on histidine to the N side. Further work will be required to decipher which other amino acid residues participate in gating functions. Our histidine switch model can be integrated to the proton pumping mechanism proposed by Sazanov and colleagues (Sazanov 2023), in which protons are transferred back-and-forth between the KE pair and the central lysine residues of the antiporters, which in the case of NuoL can be achieved with the histidine switch dynamics. In the models proposed by Kaila and colleagues (Kaila 2018), proton transfer between conserved KE pairs and central lysine residues does not occur (and also not in between the antiporter-like subunits), therefore, in this regard our model differs from the latter mechanism. Further work will be required to resolve these issues.

**Supplementary files**

Supplementary file 1 – Snapshot from AWH simulation corresponding to histidine in A conformation (see Fig. 5A, PDB format)

Supplementary file 2 – Snapshot from AWH simulation corresponding to histidine in B conformation (see Fig. 5B, PDB format)

**References**

Djurabekova, A., O. Haapanen and V. Sharma. 2020. Proton motive function of the terminal antiporter-like subunit in respiratory complex I. *Biochimica et Biophysica Acta (BBA) - Bioenergetics* 1861.

Djurabekova, A., J. Lasham, O. Zdorevskyi, V. Zickermann and V. Sharma. 2024. Long-range electron proton coupling in respiratory complex I — insights from molecular simulations of the quinone chamber and antiporter-like subunits. *Biochemical Journal* 481:499–514.

Kaila, V.R.I. 2018. Long-range proton-coupled electron transfer in biological energy conversion: towards mechanistic understanding of respiratory complex I. *Journal of The Royal Society Interface* 15.

Lee, Y., O. Haapanen, A. Altmeyer, W. Kühlbrandt, V. Sharma and V. Zickermann. 2022. Ion transfer mechanisms in Mrp-type antiporters from high resolution cryoEM and molecular dynamics simulations. *Nature Communications* 13.

Parey, K., J. Lasham, D.J. Mills, A. Djurabekova, O. Haapanen, E.G. Yoga, H. Xie, W. Kühlbrandt, V. Sharma, J. Vonck and V. Zickermann. 2021. High-resolution structure and dynamics of mitochondrial complex I—Insights into the proton pumping mechanism. *Science Advances* 7.

Pecorilla, C., A. Altmeyer, O. Haapanen, Y. Lee, V. Zickermann and V. Sharma. 2025. Conformational dynamics of a histidine molecular switch in a cation/proton antiporter. *Biochimica et Biophysica Acta (BBA) - Bioenergetics* 1866.

Sazanov, L.A. 2023. From the ‘black box' to ‘domino effect' mechanism: what have we learned from the structures of respiratory complex I. *Biochemical Journal* 480:319–333.
